# Built environment and residential blocks carbon emissions: a study using advanced metering infrastructure data

**DOI:** 10.3389/fpubh.2025.1645402

**Published:** 2025-08-14

**Authors:** Xiaoping Zhang, Zixuan Cui, Chaoxian Feng, Xin Wen, Huabin Xiao, Jianbo Ni

**Affiliations:** ^1^College of Architecture and Urban Planning, Tongji University, Shanghai, China; ^2^School of Architecture and Urban Planning, Shandong Jianzhu University, Jinan, China

**Keywords:** built environmental factors (BEF), residential blocks carbon emissions (RBCE), Random Forest model, influence mechanism, Tianjin

## Abstract

To address the pressure of emissions reduction in urban residential blocks (RBs), this study takes 99 micro-scale RBs in Hongqiao District, Tianjin as the objects, aiming to reveal the driving mechanism of built environmental factors (BEF) on residential blocks carbon emissions (RBCE) and explore planning strategies that balance carbon reduction and health benefits. By integrating spatial statistical analysis and high-precision machine learning models, the system has systematically revealed the spatio-temporal evolution laws, spatial differentiation characteristics and driving mechanisms of BEF on RBCE. Key findings include: (1) From 2021 to 2023, both the RBCE, residential blocks carbon emissions intensity (RBCEI), and average household carbon emissions (RBCE-AH) showed a “first rise then fall” fluctuation, with an overall 5.7% increase, signaling sustained emissions reduction pressure. (2) High emissions areas are spatially concentrated and contagious, while low carbon units are mostly peripheral. Spatial autocorrelation analysis indicates a significant positive correlation and a west-south clustering pattern. (3) Land area (LA) is the main emissions affecting factor, followed by green space ratio (GSR) and Land use mixing degree (LMD), whose inhibitory effect exceeds that of traditional high-intensity development indicators. (4) Targeted planning strategies such as strictly controlling land use expansion, improving GSR, and promoting functional combination were proposed. At the same time, it was suggested that in the future, the heterogeneity of building types and more three-dimensional morphological indicators should be incorporated into the BEF index system, and combined with more refined coupling models, their influence paths should be quantitatively analyzed. These strategies not only provide a basis for the implementation of macro emissions reduction policies, but also offer solutions for micro action plans centered on residents’mental health and cardiopulmonary system protection. Overall, this study provides a scientific basis for low carbon RBs planning and renewal that balances carbon reduction with health benefits.

## 1 Introduction

Greenhouse gas (GHG) emissions from human activities have exacerbated global warming, leading to various serious environmental problems and becoming a key driving factor threatening public health. According to the data released by the Intergovernmental Panel on Climate Change (IPCC), cities are home to over 55% of the global population and contribute approximately 75% of carbon emissions (1, 2). Among them, buildings, industry, and transportation are the three major sources of urban carbon emissions. With the advancement of urbanization and the improvement of people’s living standards, building carbon emissions show a continuous growth trend (3). It is evident that carbon emissions from buildings have become a key fulcrum for the coordinated governance of climate and health. There is an urgent need to reduce carbon emissions and health risks simultaneously through low-carbon planning and renovation of existing facilities (4, 5). Residential buildings account for 58.5% of the total building area and contribute 48% to the total carbon emissions from buildings. According to the “China Energy Statistical Yearbook,” urban residential electricity demand grows at an average annual rate of 11.9%, with a continuous upward trend. As the basic spatial units of residential buildings and direct carriers of household living and consumption, residential blocks (RBs) have become a significant source of urban carbon emissions (6, 7). Therefore, studying carbon reduction measures at the RBs level is crucial for achieving carbon neutrality and health equity. RBs, as key venues for daily life, are closely tied to residents’ energy use. Multiple built environmental factors (BEF) can alter residential energy consumption by influencing the local microclimate (7), and at the same time, they are related to the health of residents. Studies indicate that residential block carbon emissions (RBCE) can vary 1.8–6 times across different BEF (6). High-emission blocks are often accompanied by higher local air pollutant concentrations. Long-term exposure significantly increases the risk of residents developing asthma, chronic bronchitis, cardiovascular diseases and neurological disorders. Additionally, high-emission blocks may further increase the psychological stress, anxiety and depression risks of residents due to factors such as the intensification of the heat island effect, noise pollution and insufficient green space, especially having a greater impact on vulnerable groups such as children, women and the older adults (8). The BEF within 500–1,000 meters of RBs has the most significant impact on RBCE (9). Therefore, to maximize the synergistic benefits of climate mitigation and health, identifying and clarifying the key impacts of BEF on RBCE has gradually become an increasingly urgent research direction. Current studies on the influencing factors of RBCE can be divided into three main types. Including socio-economic factors, microclimate factors and BEF. These studies employed a variety of methods, including field measurement and questionnaire surveys, numerical simulations, econometric models, and machine learning and spatial analysis, providing an important theoretical and empirical basis for understanding the influence mechanism of RBCE.

Firstly, socio-economic factors. Numerous empirical studies have explored the socioeconomic aspects, demonstrating that factors like age, education, income, and policies drive RBCE. Commonly used methods include the STIRPAT model and baseline regression analysis (10). For example, Zhu et al. employed a probit regression model to analyze how behavioral factors, family socioeconomic characteristics, and housing structural attributes influence energy-related behaviors. They found that non-economic factors such as environmental awareness and social norms are as important as economic incentives, with educational levels and housing characteristics significantly impacting energy usage patterns (11). Xiao et al. studied the spatiotemporal carbon emissions patterns at the RBs scale in China’s Yangtze River Delta Integration Demonstration Zone. They also examined how economic growth and housing policies affect the carbon emissions patterns throughout RBs’ life cycles, offering references for cross border ecosystem governance and regional circular economy development (12). Zen et al. used statistical methods to analyze the impact of socioeconomic factors on the carbon footprint of RBs in Malaysia’s Iskandar region. They found that household income, green attitudes, and education levels significantly influence the carbon footprint of these blocks (13). The advantages of the above-mentioned methods lie in the authenticity and reliability of the data, which can reveal the statistical correlations and relative importance among factors. They can be used to predict trends based on historical data and clearly reveal the direct and indirect impacts of socio-economic variables on carbon emissions (14). However, their limitations include high requirements for data quality and quantity, and the difficulty in obtaining micro-scale (single buildings, individual behaviors) data. This leads to a limited sample size. It mainly reveals correlations and is difficult to strictly prove causal relationships. It is difficult to precisely quantify the direct impact of physical processes (such as microclimate and building physical performance) on energy consumption. It is difficult to capture complex nonlinear relationships and spatial heterogeneity.

Secondly, microclimate factors. Studying the influence of microclimate on RBCE is another important direction. Common methods include numerical simulation and field experiments, and software includes ENVI-met and Grasshopper, etc. These studies have revealed the relationship between the built environment and carbon emissions by simulating the impact of microclimate conditions on energy consumption. For example, Ren et al. centered on Dublin, generated local climate data using the Surface City Energy and Water Balance Scheme, conducted energy simulation using the Integrated Environmental Solution Virtual Environment, and deeply analyzed the impact of microclimate on heating energy demand. It was found that the layout of buildings could significantly reduce wind speed, while both temperature and wind speed significantly affected the heating demand. Among them, the presence of trees significantly reduced the heating demand and could, respectively, reduce carbon emissions from residences and apartments by 3.1 and 4.6% (15). Wei et al. used Phoenics and Ecotect software to simulate the microenvironments of Chinese RBs, analyzing the impact of microclimates on RBCE (16). Xu et al. assessed the adaptability of mountainous settlement planning in Chongqing, constructing a 3D coupling model based on heat, wind, humidity, and light, and proposed a low-carbon ecological RBs planning framework (17). Cui et al. combined field measurements with ENVI-met simulations to analyze microclimate and energy consumption optimal conditions for carbon reduction in cold region RBs (18). The advantage of the above-mentioned methods lies in its ability to deeply understand the physical mechanisms and processes, and to precisely quantify the influence of physical factors (such as the performance of the envelope structure, shading, and ventilation). The design scheme can be evaluated in the absence of actual operational data (such as during the design stage). However, the calibration of input parameters relies on actual measurement and has high computational costs. It is usually difficult to fully integrate complex human behaviors and socio-economic factors, and the simulation accuracy of actual building performance and resident behavior is limited.

Thirdly, BEF. Studying the impact of BEF (such as urban road networks, land use and architectural forms) on RBCE has been an important direction in recent years. Common methods include ordinary least squares models, geoweighted regression, energy consumption simulation and machine learning. For example, Su et al. modeled 81 residential communities geometrically, calculating lifecycle carbon emissions to analyze how layout, building height, spacing, and orientation temporally affect RBCE (9). Liao et al. used grey correlation analysis and the general global optimization method to assess building environmental factors’ impact on Zibo City’s RBCE, finding these factors significantly influence RBCE and that optimized urban design can reduce them (19). Li et al. applied geographically weighted regression to establish local regression models, revealing that BEF, public transport infrastructure, and urban road areas per capita affect RBCE, with impacts varying by location (20). Luo et al. combined optimized backpropagation neural networks with spatial weight matrices to model land use and RBCE. They found mixed commercial and residential land expansion positively impacts RBCE intensity and analyzed future RBCE spatial patterns at a 1 km resolution (21). The advantages of the above-mentioned methods lie in their powerful ability to handle complex nonlinear relationships, their skill in mining hidden patterns from massive amounts of data, their high prediction accuracy, and their ability to quantitatively assess the long-term impact of building forms on carbon emissions. However, their interpretability is usually poor, making it difficult to understand the underlying physical mechanisms. Meanwhile, most research on machine learning methods still takes administrative regions as the basic unit and lacks detailed characterization of micro-scale residential areas.

Therefore, the high-precision machine learning model and spatial statistical analysis method adopted in this study have significant advantages in terms of data processing capacity, model complexity, and result interpretability. Firstly, machine learning models can capture complex nonlinear relationships and spatial heterogeneity, making up for the limitations of traditional statistical models. Secondly, spatial statistical analysis methods can quantitatively assess the spatial dependence and dynamic changes of built environmental factors on carbon emissions, providing a scientific basis for policy-making. Finally, by integrating State Grid data and high-resolution spatial data, high-precision prediction of RBCE is achieved, deeply revealing the spatial differentiation pattern and providing a scientific basis for the planning and update of low-carbon and healthy RBs.

In recent years, with the large-scale construction of the national smart grid and the extensive installation and use of intelligent sensing devices, especially the popularization of the advanced metering infrastructure (AMI), power supply companies have obtained an extremely large amount of data with geographical identifiers and time information (22). Unlike traditional data such as survey questionnaire sampling and model simulation, AMI data can accurately and in real time reflect the spatial–temporal information of each power consuming unit, including desensitized and anonymized user names, user ownership, power consumption locations, as well as electricity consumption, voltage, power factor, etc. It has the characteristics of high accuracy, high timeliness and high coverage (23). Therefore, studies based on AMI data has gradually become an emerging research hotspot (24, 25). Institutions such as IBM, Oracle, and HP have successively released white papers on big data for smart grids, describing and envisioning the application scenarios of AMI data. EPRI has carried out a five-year scientific research project on power big data and focused on developing a power big data platform. Institutions such as PG&E, Oncor, and UCLA have also conducted research on the application of AMI data in areas such as user electricity consumption behavior analysis and classification, load forecasting, power grid planning, assessment and early warning of distribution network operation status, and optimal operation strategies, achieving some goals and results that could not be realized under previous data conditions (26). However, few systematic studies on RBCE have been conducted using AMI data. Whether the research results of AMI data support traditional data and whether deeper research conclusions can be drawn remain worthy of further exploration (19, 27).

To fill the existing research gap, this study is based on the AMI micro-scale residential area data of State Grid. By adopting a method framework that couples high-precision machine learning models and spatial statistical analysis, it has significant advantages in data processing capacity, model complexity, and result interpretability. It overcomes the limitations of traditional research methods in terms of data processing capacity, model complexity and result interpretability, and simultaneously realizes the fine identification of the spatio-temporal evolution and agglomeration patterns of RBCE on 3,564 panel data of 99 settlements. Robust estimation of nonlinear threshold effects of built environmental elements Rapid simulation of the emission reduction effect of planning scenarios. This methodological system not only makes up for the insufficiency of measured samples but also avoids the assumption bias of spatial effects in traditional econometric models, providing a directly implementable decision-making tool for micro-scale low-carbon community planning. It provides a new perspective and methodological reference for RBCE study. Firstly, we will collect and calculate carbon emissions data for 99 RBs in Tianjin’s Hongqiao District from 2021 to 2023. Secondly, Moran’s I based spatial autocorrelation analysis will be used to explore the spatial distribution characteristics of carbon emissions. Thirdly, relevant analysis models and Random Forest models will be established to examine how BEF drive carbon emissions in RBs. This study focuses on three key questions. (1) What are the spatial distribution characteristics of RBCE based on AMI data? Is there year to year differences in RBCE? (2) How do BEF influence RBCE? (3) What effective policy suggestions can be derived from the analysis for highly urbanized regions? This study aims to assist urban planners and decision-makers in formulating more targeted policies to promote urban carbon reduction and public health, thereby deepening the understanding of the spatial pattern and driving factors of RBCE in the context of new data.

## 2 Materials and methods

### 2.1 Study area

**Figure 1:**
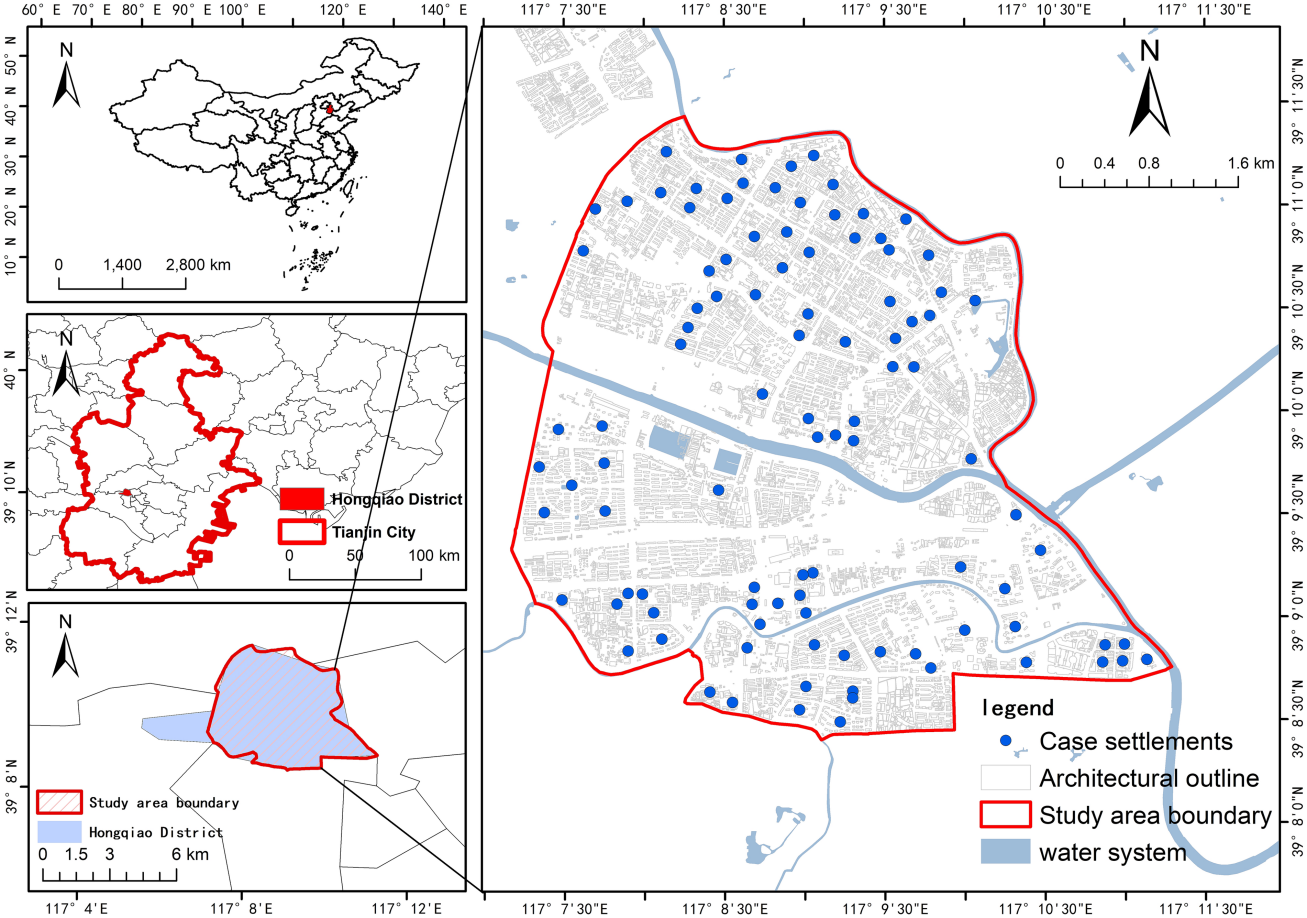
Location of study area.

Tianjin (38°34 “−40°15” N, 116°43 “−18°04” E) is in northern China. By the end of 2024, the total area of Tianjin is 11,917 km^2^, with 16 administrative districts under its jurisdiction and a permanent resident population of 13.64 million. The study area covers Hongqiao District, which is one of the six central urban districts of Tianjin. It is in the northwest of the urban area of Tianjin (Figure 1), with a jurisdiction area of 23.76 km^2^. It governs 114 RBs and has a permanent resident population of 428,800. This study selected 99 RBs in Hongqiao District as the research objects, mainly distributed in the south and north of Hongqiao District, with a relatively small distribution in the middle. At the same time, in combination with the spatial distribution of RBs, the study area is demarcated. Overall, the study area is one of the old urban districts of Tianjin, mainly composed of the old RBs. The RBCE is relatively stable and has the most significant impact on public health. Therefore, choosing Hongqiao District as the research area is typical.

### 2.2 Research data

The dataset encompasses monthly electricity consumption records for RBs in Hongqiao District, Tianjin (2021–2023), alongside BEF including land use, buildings, roads, and green spaces. Specifically, electricity consumption data were obtained from the State Grid Tianjin Electric Power Company. Land use data were sourced from the Tianjin Municipal Bureau of Planning and Natural Resources. Buildings, roads, etc. were acquired from the Tianjin Construction Bureau and open-source geospatial platforms (e.g., OpenStreetMap) (Table 1).

**Table 1:** Data sources and description.

Name	Description	Sources
Electricity consumption data	Electricity consumption data of RBs	Tianjin power supply company Public Security Bureau
Road systems of Tianjin	Vector data of road traffic system	OpenStreetMap (OSM)
Land use map of Tianjin in 2021	Master Plan of Tianjin Territorial Space	Natural Resources Bureau
Buildings	Building area, height, type, etc.	Construction Bureau
Population	The number of households of RBs	Anjuke website
Public charging piles	Location of public charging piles	Gaode map
POI	Location of public service facilities, educational service facilities, and public transportation stops	Baidu map
Remote sensing data	The coverage rate of trees	https://www.gscloud.cn/

This study employs 2021–2023 micro-scale data for three primary reasons. Firstly, 2021 marked the inaugural year of China’s formal integration of “dual carbon” goals into its national strategy, with Tianjin implementing consequential emissions reduction policies (e.g., Tianjin Carbon Peak Implementation Plan). The data of this period can keenly capture the immediate response of RBCE under policy intervention and reveal the typical characteristics of the transition period. Secondly, energy consumption data at the RB level relies on refined statistics (such as individual metering by power companies and property management ledgers). However, it was not until 2021 that Tianjin began to require each sub-district at the district level to submit energy ledgers at the RB level. Before that, Hongqiao District had neither a sub-item energy consumption ledger at the residential area scale nor made the corresponding geographic information public. Therefore, it is impossible to systematically construct microscale panel data consistent with this study. Thirdly, although the sample period is relatively short, there are already monthly data from 99 RBs for three consecutive years, totaling 3,564 sets of observations tracking the changes in carbon emissions. This is a relatively rich sample in micro-scale carbon emissions research and can partially alleviate the bias caused by the short time period. Crucially, RBCE exhibit seasonal periodicity while maintaining interannual stability. This consistency is corroborated by Zhang et al., who compared monthly electricity consumption in seven mature RBs against annual totals across 3 years (28). Consequently, RBCE patterns remain relatively stable within defined periods, establishing a well-founded basis for analysis. While extended temporal coverage would enhance robustness, the 2021–2023 period represents the most complete, comparable, and spatially verifiable RB-level sequence currently accessible through official channels. We will continue tracking data updates to test long-term effects in subsequent research.

Tianjin, located in a cold region, has seen most existing studies on RBCE focus on heating energy, with less attention given to electricity consumption (29). This study chooses electricity consumption as the research focus for two main reasons. On one hand, urban buildings in cold regions typically use centralized heating systems. The hot water generated by centralized heat sources is supplied to the heat needed for urban area heating through pipe networks. Heating data is usually provided by heating companies or heating stations and is supply-side data rather than demand-side data. Take Tianjin as an example. The data provided by the heating company is the thermal energy value that ensures the indoor temperature of the building in winter is above 18°C, rather than the actual thermal energy demand value of the building. On other hand, due to the limitations of heating methods, it is also very difficult to measure and obtain the actual demand values of heating energy consumption for different buildings (30). The energy consumption of building electricity is the summary of the terminal electricity consumption of each electricity user and represents the actual demand value. Moreover, under the same geographical location, fixed built environment and unified climatic background, the influence mechanism of the external spatial environment on the energy consumption of building electricity and heating is the same (29, 31). Therefore, in this study, the energy consumption of building electricity is selected as the basis for calculating RBCE, which can better reflect the impact of the BEF on the overall energy consumption demand of buildings. Secondly, as the economic development level of cities in cold regions, the living standards of residents and their demands for comfort continue to rise, the stock of buildings and the number of internal electrical equipment are constantly increasing. The use of air conditioners in summer and electric heating equipment in transitional seasons also significantly increases, which in turn leads to a rapid rise in the total amount of electricity consumption. Therefore, in addition to heating energy consumption, more attention should also be paid to electricity conservation in residential buildings in cold regions.

### 2.3 Methods

**Figure 2:**
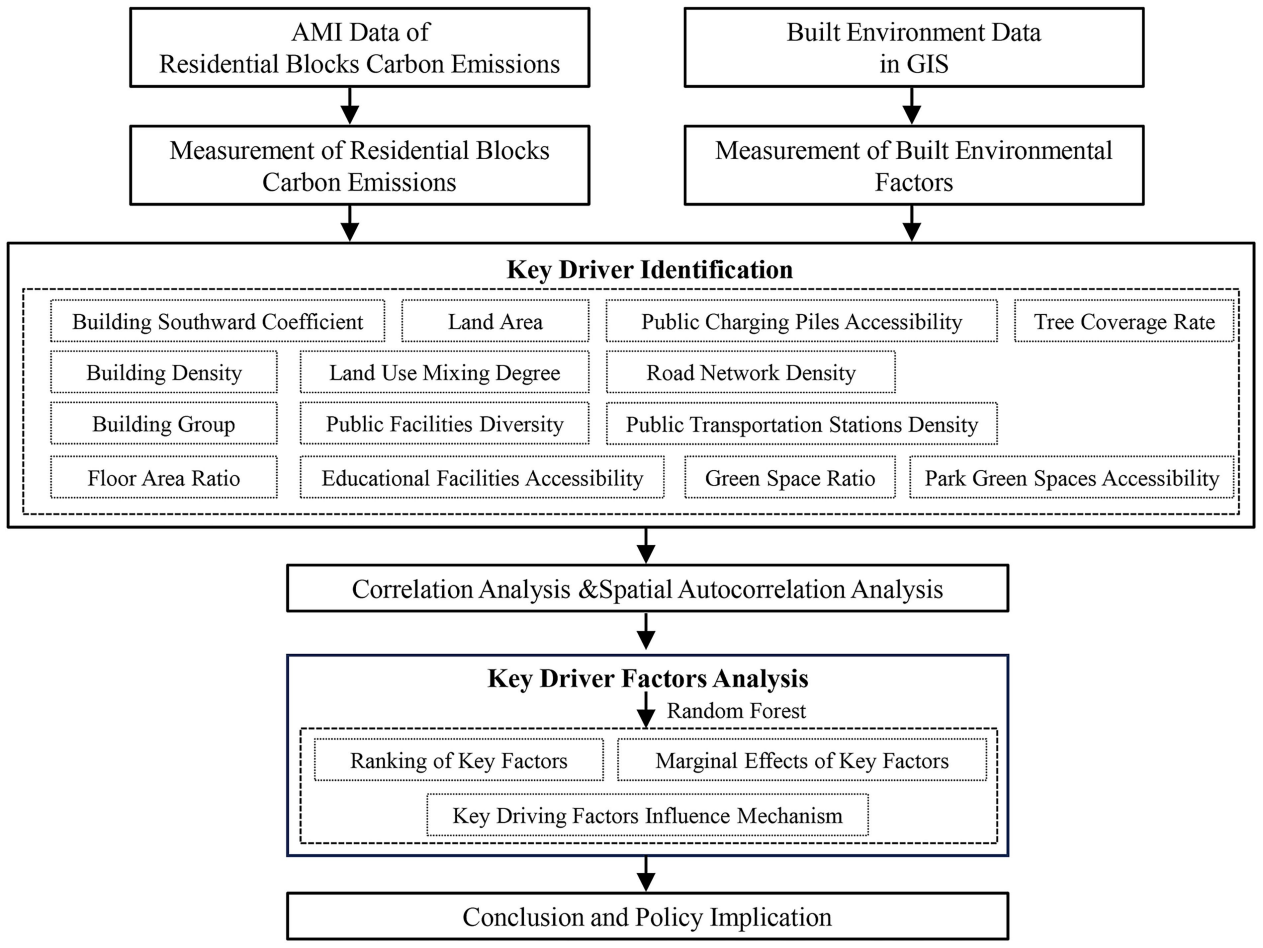
Framework of methods.

Reducing RBCE is one of the important measures to achieve the “dual carbon” goals. Due to the different BEF in different areas of the city, there may be spatial differences in RBCE, and different spatial patterns may form over time. The RBCE mainly depend on the level of economic development, the structure of energy consumption, and the degree of technological progress. However, internal factors of the block such as floor area ratio and green space ratio, as well as peripheral factors such as road network density, land mix degree, diversity of public service facilities, and accessibility of educational service facilities, will also affect RBCE intensity through various energy usage patterns and economic activities. The impact of these factors on RBCE may vary at different levels. To reveal the spatial–temporal variation patterns of RBCE and identify their key influencing factors, this study first calculated the total RBCE, residential blocks carbon emissions intensity (RBCEI) and BEF. Then, by using the spatial autocorrelation method, the spatial-temporal characteristics of the overall pattern of RBCE were analyzed. On this basis, the key factors influencing the RBCE were determined based on correlation analysis and the Random Forest model, and the linear or nonlinear response relationships between these factors and RBCE were explored. Finally, the influence mechanisms and policy applications of these key influencing factors are analyzed (Figure 2).

#### 2.3.1 Measurement of residential blocks carbon emissions

The energy consumption of RBs mainly includes buildings, transportation, waste treatment, etc. Among them, the proportion of building energy consumption is the largest. The actual energy consumption of buildings includes centralized heating, electricity and gas, which are mainly used for heating, cooling, lighting, and equipment, etc. Studies have also confirmed this. Zhang et al. collected the RBCE in Changxing, Zhejiang Province, China, and found that carbon emissions from electricity consumption were the main source of emissions, accounting for more than 98%, while carbon emissions from the consumption of natural gas accounted for only about 1% (32). Therefore, in this study, the main feature of RBCE is carbon emissions from electricity consumption. According to the carbon emission coefficient, taking the electricity consumption of each RB as the unit, the RBCE is calculated by Equation 1:


(1) E=AD×EF

where E is carbon emissions (kg), AD is electricity consumption, and EF is the carbon emissions coefficient. Among them, AD is the annual electricity consumption of residential land provided by the Tianjin power supply company. EF uses the average carbon emissions per unit of electricity consumption in North China and the value is 0.5834 kgCO_2_/kWh. Additionally, residential blocks carbon emissions intensity (RBCEI) is the ratio of RBCE to the RB area. The average household carbon emissions (RBCE-AH) is the ratio of RBCE to the number of households in the RB. The measurement method of RBCEI and RBCE-AH are defined as follows (Equation 2):


(2) I=E/S

where I is carbon emissions intensity (kg/m^2^), E is carbon emissions (kg), and S is the area of residential land (m^2^) (Equation 3).


(3) A=E/H

where A is average household carbon emissions (kg/h), E is carbon emissions (kg), and H is the number of households in the RB (h).

#### 2.3.2 Measurement of built environmental factors

According to the control requirements of Master Plan of Tianjin Territorial Space, combined with relevant literature research and through expert interviews, According to the control requirements of Master Plan of Tianjin Territorial Space, combined with relevant literature research and through expert interviews, Meanwhile, to avoid the interference of microclimate factors such as solar radiation acquisition, natural ventilation potential and thermal environment comfort within the RB, this study only uses the planar BEF attributes to describe the BEF characteristics of RBs, and does not include three-dimensional parameters such as building height, volume and sky view factor (SVF). Ultimately, The Building Southward Coefficient (BSC), Building Density (BD), and Building Group (BG) are proposed from five dimensions: architectural spatial layout, land use, facility configuration, road traffic, and ecological construction. Floor Area Ratio (FAR), Land Area (LA), Land Use Mixing Degree (LMD), Public Facilities Diversity (PFD) Educational Facilities Accessibility (EFA), Public Charging Piles Accessibility (PCPA) Road Network Density (RND), Public Transportation station Density (PTSD), Green Space Ratio (GSR) Tree Coverage Rate (TCR), Park Green Spaces Accessibility (PGSA) 14 BEF. The measurement methods of the 14 BEF can be found in Table 2.

**Table 2:** Evaluation index system of built environment.

Type	BEF	Formula	Description	Source
Architectural spatial layout (ASL)	Building southward coefficient (BSC)	*I*= $\frac{1}{n}\sum_{i=1}^{n}\frac{\mathrm{Di}}{\mathrm{Li}}$ (*i* = 1,2,…,n)	I represent the southward coefficient index of residential buildings; n represents the number of individual buildings; Di represents the maximum projected length of the i-th building in the east–west direction. Li is the perimeter of the i-th building.	(47)
	Building density (BD)	$S=\sum_{i=1}^{n}\frac{s_{i}}{s}$ (*i* = 1,2,…n)	S represent the building density; n represents the number of individual buildings; $s_{i}$ represent the base area of the i-th building, and S represents the total land area.	(48)
	Building group (BG)	*Z* = Xi (*i* = 1,2,…5)	Z represent the architectural combination form; Xi represents the first type of architectural combination form. 1 is a standalone building, 2 is a scattered building, 3 is an enclosed building, 4 is a row building, and 5 is a combined building.	(49)
Land use (LU)	Floor area ratio(FAR)	*F*= $\sum_{i=1}^{n}\frac{M_{i}}{s}$ (*i* = 1,2,…,n)	F represent the floor area ratio; n represents the number of individual buildings; $M_{i}$ represents the total area of the i-th building. S represents the total land area.	(45)
	Land area(LA)	--	LA refers to the total land area of the RB, which is directly calculated and extracted through the GIS system.	(50)
	Land use mixing degree(LMD)	*T* = (−1) $\sum_{i=1}^{n}(\frac{\mathrm{Li}}{u})\ln(\frac{\mathrm{Li}}{u})$ (*i* = 1,2,…,n)	T represents the degree of land mix in RB; u represents the total number of POI within a 1-kilometer radius of the RB. n represents the total number of POI classes within the range; Li represents the quantity of the i-th type POI within the range.	(51)
Facility configuration (FC)	Public facilities diversity(PFD)	*C*= $\sum_{i=1}^{n}\frac{\mathrm{Li}}{S}$ (*i* = 1,2,…,n)	C represents the diversity of public service facilities; S represents an RB of 1 square kilometer. n represents the total number of types of public service facilities within the range; Li represents the number of public service facilities of Category i within the scope.	(52)
	Educational facilities accessibility(EFA)	*Q* = Min(Li) (*i* = 1,2,…,n)	Q is the accessibility of educational service facilities; The length of the travel route from the Li Type i RB to the corresponding primary school in the school district.	(53)
Road traffic (RT)	Public charging piles accessibility(PCPA)	*M* = Min(Li) (*i* = 1,2,…,n)	M represents the configuration density of public charging piles; The length of the travel route from the Li Type i residential area to the surrounding public charging stations.	(54)
	Road network density(RND)	*K*= $\sum_{i=1}^{n}\frac{\mathrm{Li}}{S}$ (*i* = 1,2,…,n)	K represents the road network density within a 1-kilometer radius of the RB. n represents the number of roads within the range; Li represents the length of the i-th road within the range; S represents the area within 1 meter of the RB.	(55, 56)
	Public transportation stations density(PTSD)	*D*= $\frac{R}{S}$	D represents the density of public transportation stops; R represents the number of public transportations stops within a 1-kilometer radius of the RB. S represents the area within a 1-kilometer radius of the RB.	(57)
Ecological construction (EC)	Green space ratio(GSR)	*G*= $\sum_{i=1}^{n}\frac{s_{i}}{S}$ (*i* = 1,2,…,n)	G represents the green space ratio; n represents the total number of green spaces within the RB; $s_{i}$ represents the area of the i-th green space within the RB; S represents the total land area of the RB.	(58)
	Tree coverage rate(TCR)	E= $\frac{G}{S}$	E represents the coverage rate of trees in the RB; G represents the projected area of the canopies of all the trees in the RB on the ground. S represents the total land area of the RB.	(59)
	Park green spaces accessibility (PGSA)	*W* = Min(Li) (*i* = 1,2,…,n)	W represents the accessibility of the park green space in the RB; The length of the travel route from the Li Type i RB to the surrounding parks and green spaces.	(60)

#### 2.3.3 Pearson correlation analysis

This study conducts correlation analysis through the Pearson correlation coefficient. In statistics, the Pearson correlation coefficient can be used to measure the correlation between two variables X and Y. Its value ranges from −1 to 1, describing the strength of the linear correlation between the two variables. The larger its absolute value, the stronger the correlation. The measurement method of Pearson correlation coefficient is defined as Equation 4:


(4) $$r=\frac{1}{n-1}\sum_{i=1}^{n}(\frac{X_{i}-\bar{X}}{S_{X}})(\frac{Y_{i}-\bar{Y}}{S_{Y}})$$

Where r is the Pearson correlation coefficient, X and $S_{X}$ are the sample mean and sample standard deviation, respectively.

#### 2.3.4 Spatial autocorrelation analysis

Spatial autocorrelation analysis is mainly used to describe the spatial distribution characteristics of variables, and it includes global and local spatial autocorrelation analysis. The global Moran’s I describes the degree of agglomeration in space, and its value range is [−1, 1]. A Moran’s I greater than zero indicates a positive correlation in the spatial distribution of carbon emissions, and the closer it is to 1, the higher the spatial agglomeration of carbon emissions. A Moran’s I less than zero indicates that the spatial distribution of carbon emissions is different. The closer it is to −1, the stronger the spatial heterogeneity of carbon emissions. A Moran’s I equal to zero indicates that carbon emissions in the study area are not correlated. The measurement method of Moran’s I are defined as follows (Equations 5, 6):


(5) $$I=\frac{n\sum_{i=1}^{n}\sum_{j=1}^{n}W_{\mathrm{ij}}(y_{i}-\bar{y})(y_{j}-\bar{y})}{S^{2}\sum_{i=1}^{n}\sum_{j=1}^{n}W_{\mathrm{ij}}}$$

(6) $$S^{2}=\sum_{i=1}^{n}{\left(y_{i}-\bar{y}\right)}^{2},\bar{y}=\frac{1}{n}\sum_{i=1}^{n}y_{i}$$

Where I represents the Moran’s I, n is the total number of spatial units in the study area, $y_{i}$ and $y_{j}$ respectively represent the mean carbon emissions of the i-th and j-th spatial units, and $W_{\mathrm{ij}}$ is the standardized spatial weight matrix. The spatial weight matrix constructed in this study adopts the Queen adjacency matrix, that is, if there are common edges or common vertices between regional unit i and regional unit j, then $W_{\mathrm{ij}}=1$ ; Otherwise, $W_{\mathrm{ij}}=0$ .

The global Moran’s I can prove whether the spatial autocorrelation between regional elements is significant or not through the assumption of normal distribution. The spatial autocorrelation is tested based on the standardized *Z* value and *p* value. The specific test rule is as follows: If |*Z*| ≤ 1.96, *p* > 0.05, that is, there is no spatial autocorrelation of RBCE among regional units. If |*Z*| ≥ 1.96 and *p* ≤ 0.05, the spatial autocorrelation of RBCE among regional units is significant. If |*Z*| ≥ 2.58 and *p* ≤ 0.01, the spatial autocorrelation of RBCE among regional units is extremely significant. When the *Z* value is greater than 0 and reaches the significance level, it indicates that the RBCE among regional units present an aggregated distribution, that is, the high (low) RBCE areas are adjacent to the high (low) RBCE areas. When the *Z* value is less than 0 and reaches the significance level, it indicates that the RBCE among regional units present a dispersed distribution, that is, high (low) RBCE zones and low (high) RBCE zones are distributed alternately. The measurement method of the *Z* value are defined as follows (Equations 7, 8):


(7) $$Z(I)=\frac{I-E(I)}{\sqrt{\mathrm{VAR}(I)}}$$

(8) $$E(I)=\frac{1}{n-1}$$

Where E(I) represents the expected value of the global Moran’s I, and $\sqrt{\mathrm{VAR}(I)}$ represents the theoretical variance of the global Moran’s I.

Global spatial autocorrelation analysis explores the spatial agglomeration degree of RBCE from the perspective of the overall study area and determines whether there is spatial correlation of RBCE. However, to a certain extent, it ignores the atypical spatial characteristics existing in the study area. Local spatial autocorrelation analysis can effectively fill this research gap. The local spatial autocorrelation analysis divides the four quadrants of HH (high-high agglomeration area), HL (high-low agglomeration area), LL (low-low agglomeration area), and LH (low-high agglomeration area) through the Moran scatter plot. Among them, HH (LL) represents that the adjacent spatial units have spatial homogeneity, and HL (LH) represents that the adjacent spatial units have spatial heterogeneity. The Moran scatter plot clearly and intuitively presents the correlation of local Spaces in the form of quadrants.

#### 2.3.5 Random Forest model

The Random Forest model, proposed by Breiman, is a machine learning algorithm based on classification trees. The Random Forest model consists of multiple decision trees, and the overall accuracy and stability are improved by combining the prediction results of these decision trees. The Random Forest model performs well in dealing with both classification and regression problems, and can handle large amounts of data and high-dimensional features. Several key characteristics of the Random Forest model: (1) Ensemble learning Random Forest is a type of ensemble learning method. It makes the final decision by constructing multiple decision trees and summarizing their results; (2) Random selection of features. When training each decision tree, the Random Forest model does not examine all possible features at each node, but randomly selects some features. This method can increase the diversity among trees and improve the generalization ability of the model; (3) Anti-overfitting performance. Because multiple decision trees are integrated, Random Forests usually have better anti-overfitting performance than a single decision tree and (4) Evaluation of variable importance. Random Forest can provide a ranking of which features are more important for the predictor variables, which is achieved by observing the contribution of feature splitting in the tree nodes to the model performance. The typical characteristics of Random Forest indicate that they are suitable for dealing with the carbon emissions of residential blocks with many characteristic variables and complex variable relationships.

Random Forest model can be implemented by using a variety of software programs, including Matlab, Python, and R languages. This study chose to build a Random Forest model based on Matlab according to data characteristics. Taking RBCE as the dependent variable and BEF at each spatial scale as the independent variable, K Bootstrap sampling was conducted on the sample set. The data was divided into the training set and the test set in a ratio of 3:7 (30% for the test set and 70% for the training set). The training set and test set are randomly allocated by the Random Forest package to ensure that the data is evenly distributed in all regions. Calculate the importance of each variable using the model accuracy (*R*^2^) of the test set.

## 3 Results

### 3.1 Characteristics of RBCE

#### 3.1.1 Quantitative characteristics

The results of RBCE and RBCEI conducted in Arcgis10.2 are shown in Table 3. In terms of RBCE, the average values of RBCE from 2021 to 2023 were 1,480,108.283, 1,676,179.839, and 156,555.401 kgCO_2_, respectively. Compared with 2021, the average RBCE in 2022 increased by 196,071.556 kgCO_2_, accounting for 13.25% of the average RBCE in 2021. Compared with 2022, the average RBCE in 2023 decreased by 111,124.438 kgCO_2_. It accounted for 6.63% of the average RBCE in 2022. Overall, the RBCE in 2023 increased by 84,947.118 kg of CO_2_ compared to the average RBCE in 2021, accounting for 5.74% of the average RBCE in 2021. Additionally, from 2021 to 2023, the highest RBCE was achieved by Jie Yuan (ranked 99th), with carbon emissions of 5,833,847.875, 6,533,121.082, and 6,043,520.427 kgCO_2_, respectively. It is located in Jieyuan Sub-district in the south of Hongqiao District. It is an apartment complex with tower-like buildings and was completed and put into use in 2013. Therefore, it has a relatively high FAR and is also the RB with the largest FAR among all the samples. Meanwhile, from 2021 to 2023, the lowest RBCE was achieved by Yuxing Xili (ranked 40th), with carbon emissions of 84,669.935, 86,149.958, and 85,819.314 kgCO_2_, respectively. It is located in Dingzigu Sub-district in the northern part of Hongqiao District, belonging to the old urban area. It was completed and put into use in 1990 and is a multi-story old residential area.

**Table 3:** The value of RBCE, RBCEI, and RBCE-AH.

Year	Variables	Sample size	Mean	SD	Minimum	Maximum
2021	RBCE	99	2916252.930	14489653.960	84,670	146,530,720
	RBCEI	99	60.573	124.419	5.066	1251.721
	RBCE-AH	99	1640.379	7824093.122	511.063	28597.294
2022	RBCE	99	3302572.160	16409410.664	86,150	165,941,804
	RBCEI	99	68.547	139.497	5.805	1401.759
	RBCE-AH	99	1859.728	9860030.865	585.646	32025.103
2023	RBCE	99	3083624.030	15322419.826	85,819	154,940,485
	RBCEI	99	63.585	128.962	5.316	1296.709
	RBCE-AH	99	1726.470	8416393.897	536.248	29625.100

In terms of RBCEI, the average values of RBCEI from 2021 to 2023 were 60.573, 68.547, and 63.585 kgCO_2_/m^2,^ respectively. Compared with 2021, the average value of RBCEI increased by 7.974 kgCO_2_/m^2^ in 2022, accounting for 13.16% of the average RBCEI in 2021. Compared with 2022, the average value of RBCEI decreased by 4.962 kgCO_2_/m^2^ in 2023. It accounted for 7.24% of the average RBCEI in 2022. Overall, the RBCEI in 2023 increased by 3.012 kgCO_2_/m^2^ compared to the average RBCEI in 2021, accounting for 4.97% of the average RBCEI in 2021. From 2021 to 2023, the highest RBCEI was achieved by Jie Yuan (ranked 99th), with RBCEI of 1,251.721, 1,401.759, and 1,296.709 kgCO_2_/m^2^, respectively. Dingfa Home (ranked 90th) has the smallest RBCEI, which were 5.066, 5.805, and 5.316 kgCO_2_/m^2^, respectively, from 2021 to 2023. It is located in the southern part of Hongqiao District, Shaogongzhuang Sub-district, and was completed and put into use in 2002. It is mainly composed of 7-story slab residential buildings.

In terms of RBCE-AH, the highest values of RBCE-AH from 2021 to 2023 were all in Jieyuan (ranked 99th), with RBCE-AH of 28,597.290, 32,025.100, and 29,625.100 kgCO_2_/h, respectively. Jieyuan is located in Jieyuan Sub-district in the south of Hongqiao District. It was completed and put into use in 2013. It is an apartment-style RB with a tower-style building form and a relatively high FAR. There are 204 households in the RB. Meanwhile, the lowest RBCE-AH values from 2021 to 2023 were all for Dingfa Jiayuan (ranked 90th), with RBCE-AH values of 511.060, 585.650, and 536.250 kgCO_2_/h, respectively. Dingfa Jiayuan is located in the southern part of the study area in Shaogongzhuang Sub-district. It was completed and put into use in 2002. It is an old-style RB with a slab building form, 7 floors high, and a relatively low FAR. There are 492 households in the RB. Overall, the RBCE-AH was the highest in 2022 from 2021 to 2023, and the RBCE-AH has generally shown an upward trend. The RBs with higher RBCE-AH are concentrated in the western and southern parts of the study area, and the RBs with RBCE-AH increasing from low to high show an upward trend.

#### 3.1.2 Spatial characteristics

**Figure 3:**
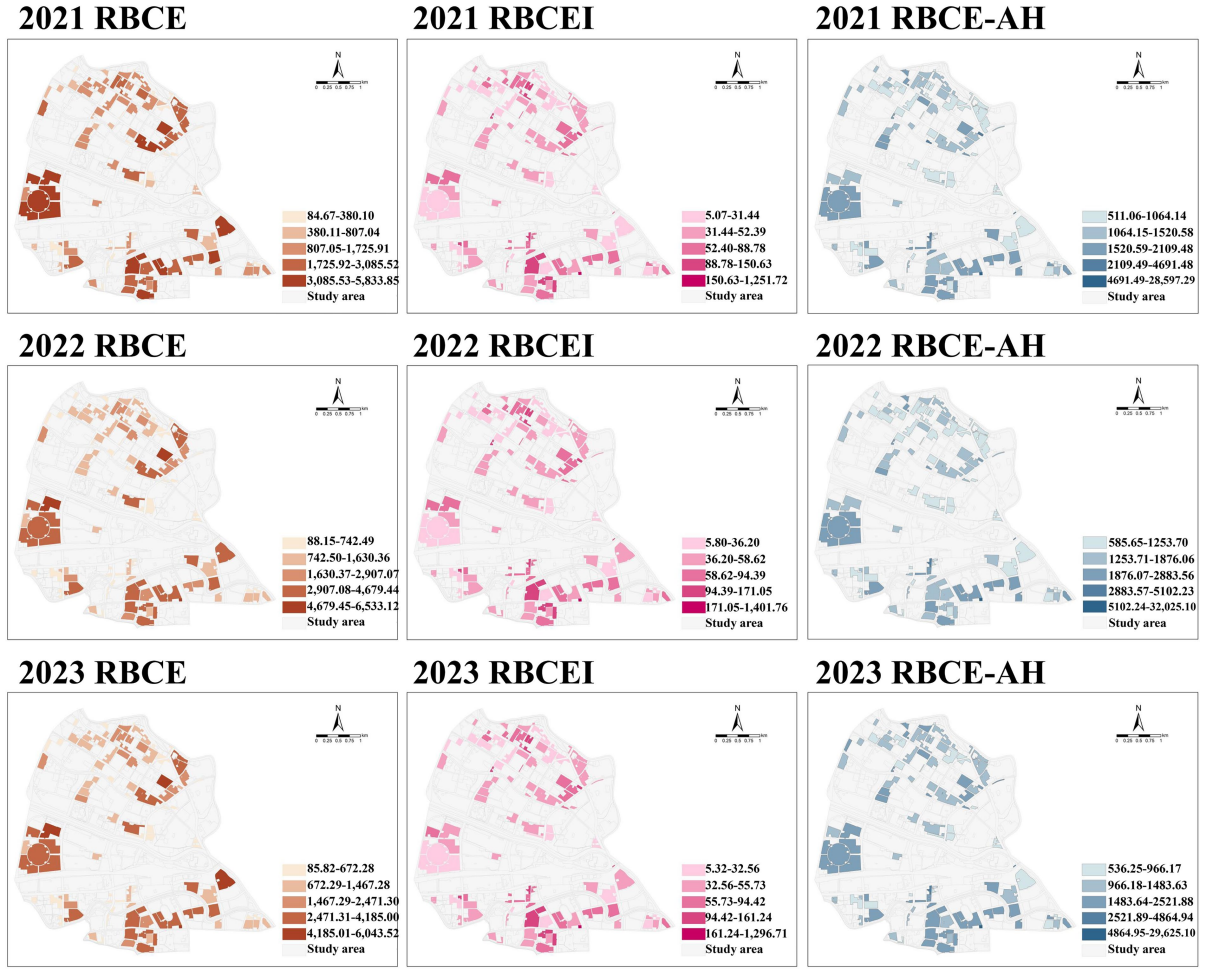
Spatial distribution of RBCE, RBCEI and RBCE-AH in 2021–2023.

The spatial distribution of RBCE and RBCEI in 99 RBs within the study area from 2021 to 2023 is shown in Figure 3. Among them, regarding the RBCE, the RBs with relatively high carbon emissions from 2021 to 2023 are mainly concentrated in Shuimutiancheng Central Garden in the east, Caifengli in the northeast, and Longchunli in the southeast. High-carbon emissions RBs are mostly concentrated and contiguous residential blocks. The RBs with relatively low carbon emissions are mainly concentrated in Qingcheng, Yongmingli, Dingfa Home and Caoyuanlou. Secondly, regarding RBCEI, the RBs with relatively strong carbon emissions intensity from 2021 to 2023 are mainly concentrated in the northern 12th Section, Shengchailou, Xingchengli, and Fanyang Building, while in the southern part, Hongliyuan, Kanghuali, Jinfeng Apartment, and Jieyuan. The RBs with relatively low carbon emissions intensity are mainly concentrated in Shuimutiancheng Central Garden, Shuixiyuan, Tianguili, Beikai Garden, Hehai Garden, Xinkaidongli, Changpingxili, Changpingli, Feiyuelou, Suizhonglou, Sanjiangli, Renheli, etc. Most of the low- carbon emissions intensity RBs are located in the peripheral areas of Hongqiao District, far from the center of the district. Thirdly, regarding RBCE-AH, The RBs with relatively high RBCE-AH from 2021 to 2023 are mainly concentrated in Jieyuan, Jinfeng Apartment, and Yifu Li in the south of Hongqiao District. Most of the RBs buildings with high RBCE-AH are high-rise RBs. The RBs with relatively low RBCE-AH are mainly concentrated in Dingfa Home, Jianshe Li, Cuishanlou, and Donglou in the north of Hongqiao District. They are mainly continuous multi-storey RBs, which are somewhat related to the development and construction model of Hongqiao District.

**Figure 4:**
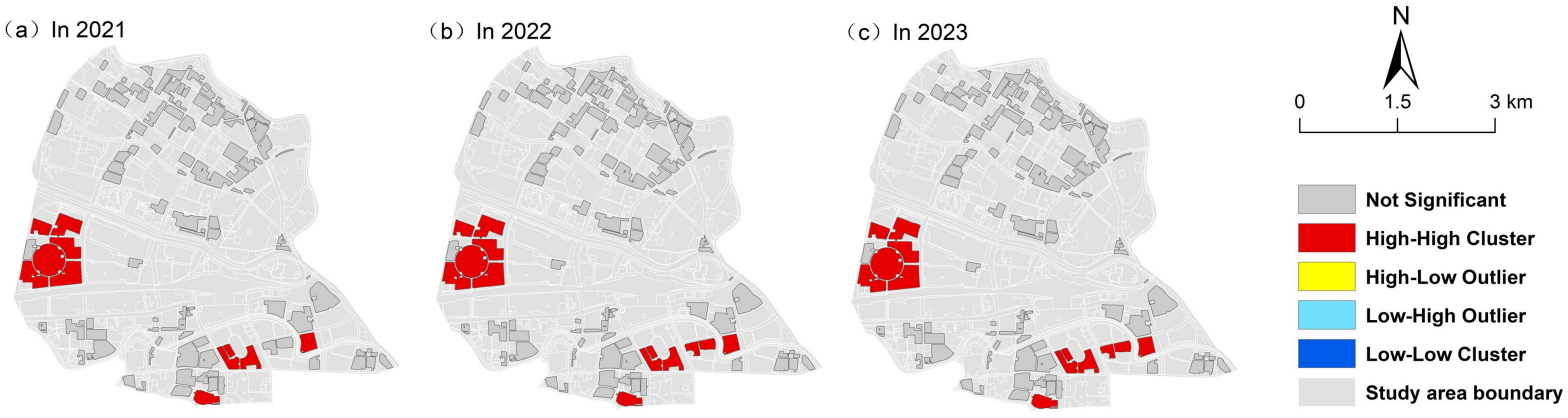
Local spatial autocorrelation of RBCE. **(a)** Local spatial autocorrelation of RBCE in 2021. **(b)** Local spatial autocorrelation of RBCE in 2022. **(c)** Local spatial autocorrelation of RBCE in 2023.

In this study, referring to the life circle theory, three spatial weight matrices with distances of 500, 1,000 and 1,500 m were constructed, and the Moran’ I of RBCE under different spatial distances was compared. Through comparison, the spatial weight matrix with a distance of 1,000 m is selected. From 2021 to 2023, the Moran’ I was 0.275, 0.291, and 0.288, respectively. The Z scores were all greater than 1.96 and *P* was all less than 0.01 (Table 4). The results indicate that RBCE shows a significant spatial positive correlation. Taking the spatial weight matrix with a distance of 1,000 m as an example, it can be seen from Figure 4 that, except for the not Significant and Neighborless types, the RBCE distribution in the four quadrants is not uniform. The spatial aggregation of RBCE is mainly high, mainly distributed on the west and south sides of Hongqiao District.

**Table 4:** Moran’I of RBCE.

Year	Moran’I	Variance	Z score	*P*-value
2021	0.275	0.002631	5.565831	0.000
2022	0.291	0.002636	5.858769	0.000
2023	0.288	0.002635	5.82356	0.000

### 3.2 Characteristics of BEF

#### 3.2.1 Quantitative characteristics

The descriptive statistics of the 14 BEF proposed in this study are shown in Table 5. All variables contain 99 samples with no missing values, reflecting BEF characteristics at different scales. The mean, standard deviation, minimum and maximum values of each BEF indicate that there are significant differences in the range of values and the degree of dispersion among different BEF. For example, the standard deviation of the LA (34,496.825) is close to its mean (38,367.342), and the minimum and maximum values are 1,291.262 and 188,360.035 respectively, indicating a significant difference in LA among different RBs. Similarly, the EFA, PCPA, and PGSA also show significant dispersion (with standard deviations reaching 51, 59, and 49% of the mean, respectively). In contrast, the variations in BD and LUMD were relatively small (standard deviations were only 9 and 7.8% of the mean, respectively) (Table 5). These statistical characteristics suggest that some variables may have skewed distributions or extreme values. In subsequent analyses, data transformation (such as logarithmic transformation) and standardization processing need to be carried out to meet the assumptions of the regression model.

**Table 5:** The value of BEF.

BEF	Sample size	Mean	SD	Minimum	Maximum
BSC	99	0.333	0.030	0.226	0.425
BD	99	0.286	0.086	0.104	0.607
BG	99	3.727	1.052	1.000	5.000
FAR	99	1.897	0.721	0.8.	5.240
LA	99	38367.342	34496.825	1291.262	188360.035
LUMD	99	2.289	0.178	1.922	2.556
PFD	99	714.017	254.518	268.255	1408.560
EFA	99	1264.586	645.615	27.000	3258.000
PCPA	99	621.586	366.709	10.000	1954.000
RND	99	9.506	2.001	6.617	14.532
PTSD	99	4.516	1.585	1.151	8.256
GSR	99	23.970	8.299	10.000	50.000
TCR	99	0.531	0.120	0.255	0.890
PGSA	99	875.949	430.863	7.000	2192.000

#### 3.2.2 Spatial characteristics

**Figure 5:**
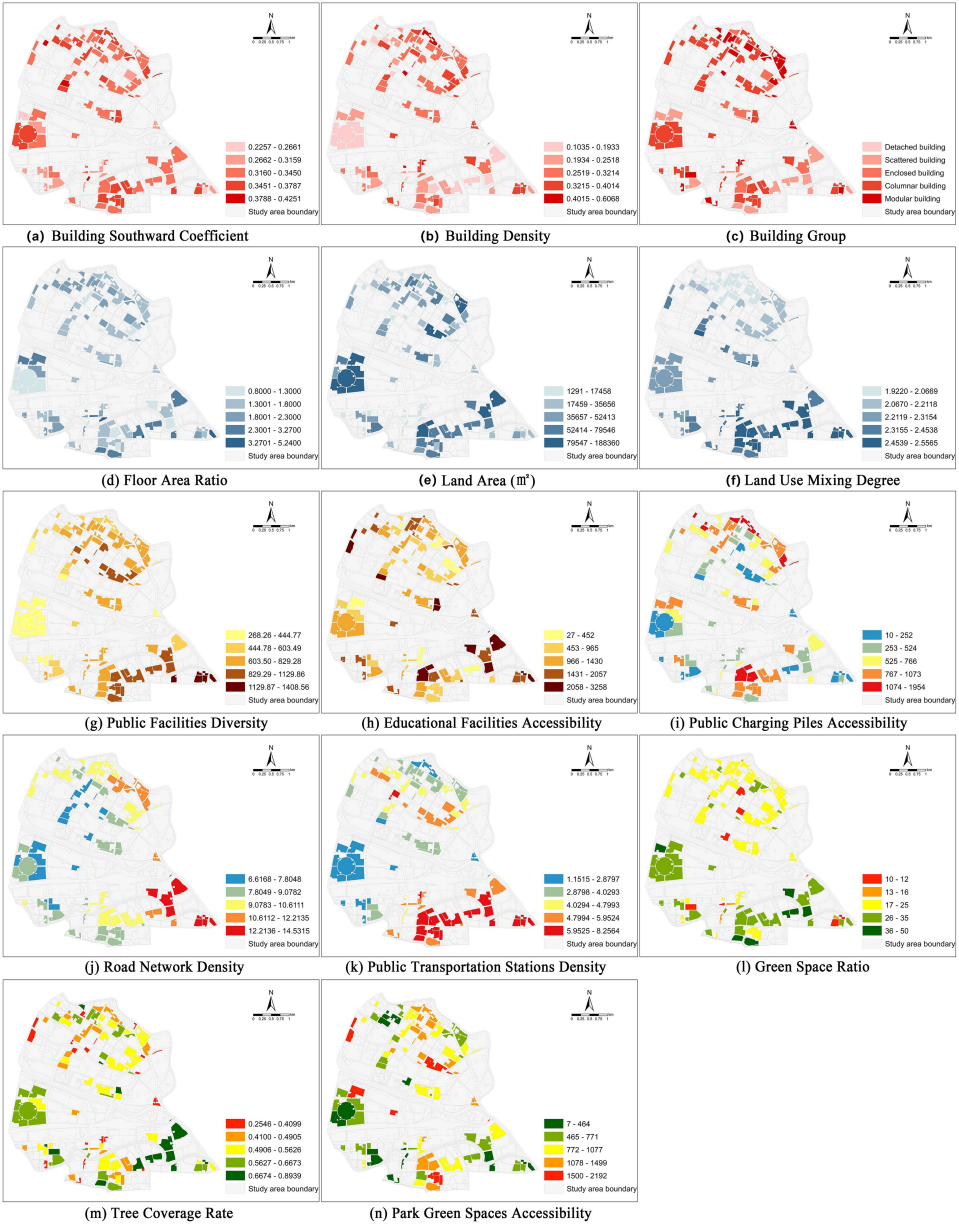
Spatial distribution of BEF. **(a)** Spatial distribution of Building Southward Coefficient. **(b)** Spatial distribution of Building Density. **(c)** Spatial distribution of Building Group. **(d)** Spatial distribution of Floor Area Ratio. **(e)** Spatial distribution of Land Area. **(f)** Spatial distribution of Land Use Mixing Degree. **(g)** Spatial distribution of Public Facilities Diversity. **(h)** Spatial distribution of Educational Facilities Accessibility. **(i)** Spatial distribution of Public Charging Piles Accessibility. **(j)** Spatial distribution of Road Network Density. **(k)** Spatial distribution of Public Transportation Station Density. **(l)** Spatial distribution of Green Space Ratio. **(m)** Spatial distribution of Tree Coverage Rate. **(n)** Spatial distribution of Park Green Spaces Accessibility.

Through data collection, the BEF of the case RBs in Hongqiao District, Tianjin were quantitatively calculated and visually expressed in Arcgis10.2. As shown in Figures 5a–c, in terms of the spatial layout of residential buildings, the BSC difference is small and evenly distributed. The difference in BD mainly lies between the north and the south. Moreover, there are a large number of high-density RBs. The BG are mostly combined and row types, and the distribution of single-family buildings is relatively small. As shown in Figures 5d–f, at the land use level, there are obvious differences in the BEF characteristics of the case RBs between the north and the south. The FAR, LA, and LMD show different performances between the north and the south. As shown in Figures 5g,h, there are relatively obvious east–west differences in the configuration of RBs facilities. The configuration of PFD performs better in the northern center and the western part of the south. The distribution of EFA is relatively balanced. Among them, the accessibility along the southeast-northwest direction is poor, and there are more high values in the southern area. As shown in Figures 5i–k, at the level of low-carbon transportation, the classification within RBs is relatively obvious. The PCPA, RND, and PTSD vary in different regions. Overall, the PTSD within the study area is relatively high near Metro Line 1. As shown in Figures 5l-n, there is a phenomenon that the ecological construction of RBs is stronger in the south than in the north. Residents in the southern RBs can obtain more green space resources in a shorter time, and the ecological construction within the RBs is also relatively better. Overall, there are significant differences in land use and low-carbon transportation among RBs within the study zone. The internal balance between the southern and northern regions is relatively balanced, but the contrast between the south and the north is more obvious.

### 3.3 The impact of BEF on RBCE

#### 3.3.1 Correlation analysis of BEF and RBCE

**Figure 6:**
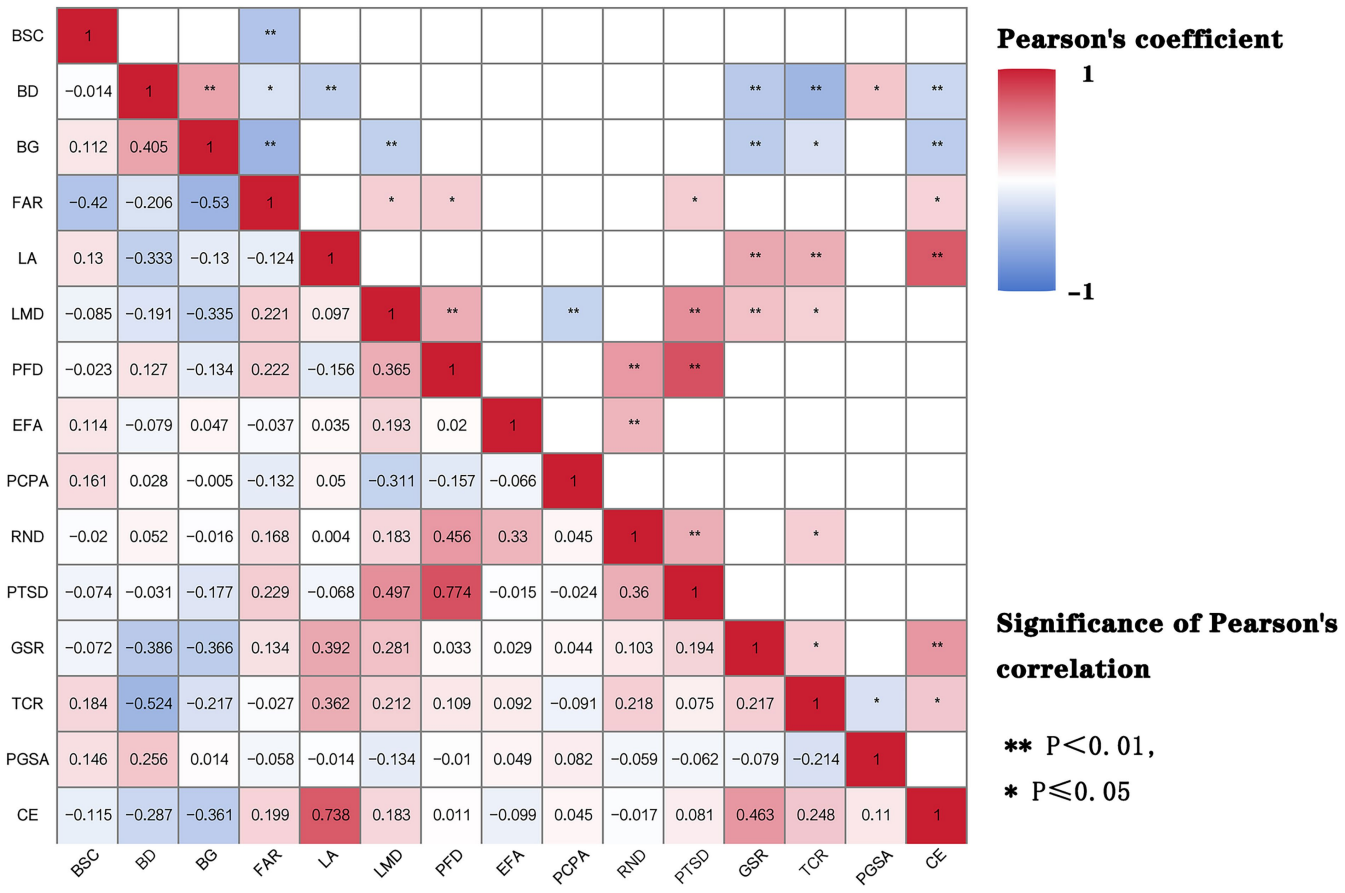
Pearson correlation coefficient between BEF and RBCE in 2023.

Based on the carbon emission data and BEF data of 99 RBs in 2023, the Pearson correlation coefficient was selected to represent the correlation intensity between RBCE and BEF, and it was determined that there was a significant correlation between BEF and different types of RBs. Among them, in the ASL, the correlation coefficients of the BSC, BD, BG, and RBCE are −0.115, −0.287** and −0.361**, respectively. The significance levels of BD and BG have passed the 0.000 test, while the significance level of the BSC has not passed the 0.05 test, indicating that There was a significant negative correlation between BD, BG and RBCE, while the correlation of the BSC was not strong. In LU, the correlation coefficients of FAR, LA, LMD and RBCE were 0.199, 0.738** and 0.183, respectively. The significance level of LA passed the 0.000 test, while the significance levels of FAR and LMD did not pass the 0.05 test, indicating that there is a significant positive correlation between LA and RBCE. Meanwhile, the FAR is positively correlated with LMD, but the significance is not strong. In the FC, the correlation coefficients between the PFD, EFA and RBCE were 0.011 and −0.099, respectively. Neither of the significance levels passed the 0.05 test, indicating that there were positive and negative correlations, respectively, between the PFD, EFA and RBCE, but the significance was not strong. In terms of RT, the correlation coefficients between PCPA, RND, PTSD, and RBCE were 0.045, −0.017, and 0.081, respectively. None of the significance levels passed the 0.05 test, indicating that there were positive correlations, negative correlations and positive correlations between PCPA, PCPA, PTSD and RBCE, respectively. But it is not significant. In terms of EC, the correlation coefficients of GSR, TCR, PGSA, and RBCE were −0.463**, −0.248**, and 0.11, respectively. The significance levels of GSR and TCR passed the 0.000 test, while the significance level of PGSA did not pass the 0.05 test. This indicates that there are significant negative correlations between GSR, TCR, PGSA and RBCE, while the correlation of PGSA is not strong. In terms of correlation, the correlation between LA and RBCE is the strongest, followed by GSR, BG, TCR, BD, FAR, LMD, BSC, PGSA, EFA, PTSD, PCPA, RND, and PFD. At the scale of RBs, the GSR has a significant impact on UBCE (Figure 6).

The Pearson correlation coefficient can only reflect the degree of linear association between two variables and cannot reveal the complex relationship under the joint action of multiple variables. Moreover, weak correlation is indeed difficult to fully describe the connection between variables. To further explore the comprehensive influence mechanism of BEF on RBCE, the study conducted a more rigorous multiple linear regression analysis as a supplement. The results show that the overall explanatory power of the regression model including all 14 BEF has significantly improved (*R*^2^ = 0.80), indicating that these factors jointly explain 80% of the variation in RBCE. The model has a high degree of goodness of fit, far superior to the previously observed simple pairwise correlations (Table 6). Meanwhile, the variance inflation factor (VIF) diagnoses for each factor were all below the critical threshold, which strongly confirmed the low degree of multicollinearity among these factors and ensured the stability and interpretability of the regression coefficient estimation (Table 7). This rigorous regression analysis result not only significantly enhanced the model’s explanatory power for RBCE, but also fully verified the independent contribution potential of the selected BEF in driving RBCE, providing a more reliable and in-depth statistical basis for understanding the complex relationship between the BEF and RBCE.

**Table 6:** The fitting of multiple regression model.

Model	*R*	*R* ^2^	Adjusted *R*^2^	Error in standard estimation	Durbin Watson
1	0.848^a^	0.718	0.671	790.8010629519	2.274

^a^Adjusted coefficient of determination (Adjusted R).

**Table 7:** Analysis of variance for multiple regression model.

Model	Sum of squares	Degree of freedom	Mean square	*F*	Significance
Regression	134012877.229	14	9572348.373	15.307	0.000^a^
Residual error	52530770.978	84	625366.321		
Total	186543648.207	98			

^a^It means that *p* < 0.05 level.

#### 3.3.2 Random Forest analysis

**Figure 7:**
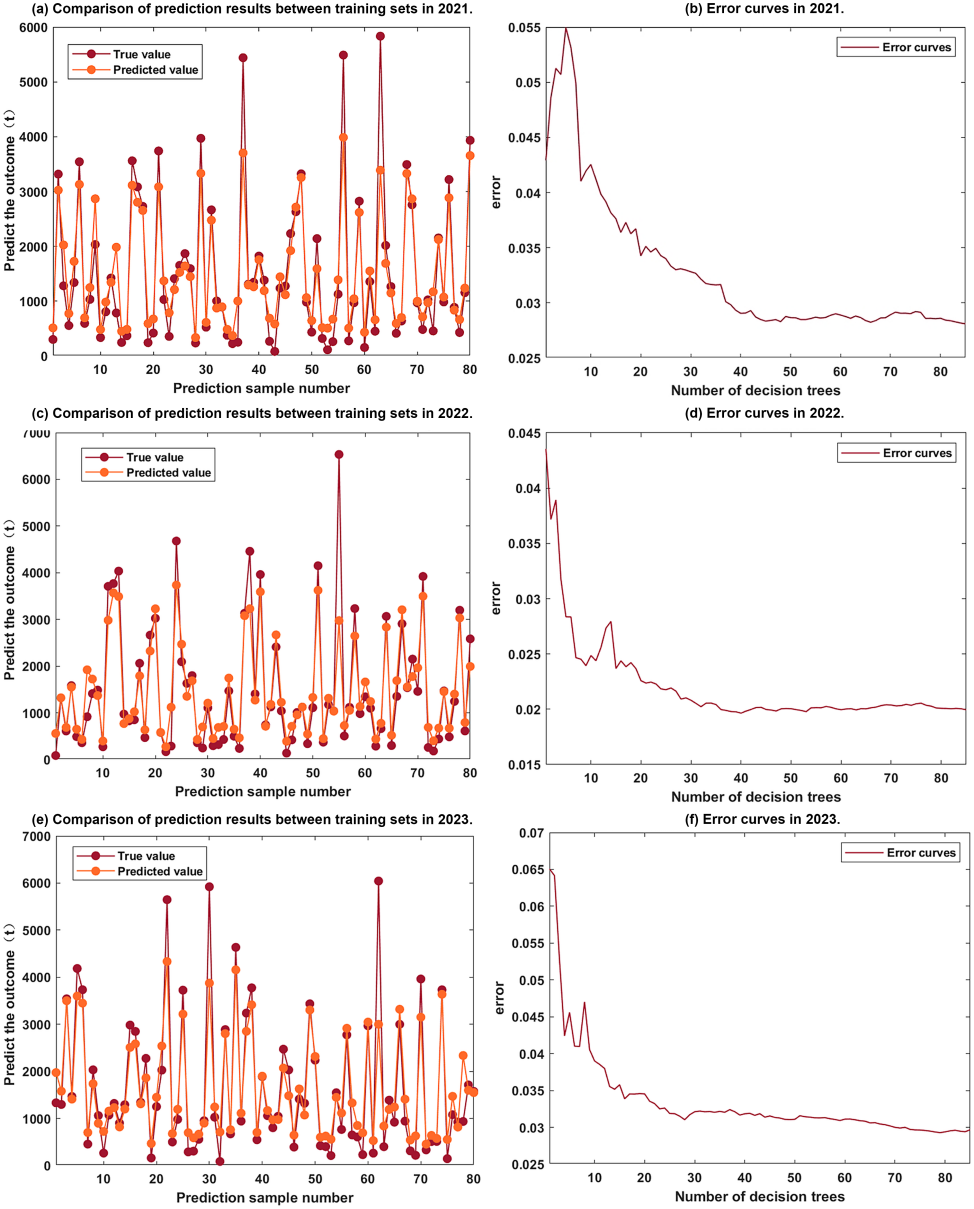
Fitting diagram of the Random Forest model. **(a)** Comparison of prediction results between training sets in 2021. **(b)** Error curves in 2021. **(c)** Comparison of prediction results between training sets in 2022. **(d)** Error curves in 2022. **(e)** Comparison of prediction results between training sets in 2023. **(f)** Error curves in 2023.

This study selects 14 BEF as characteristic data and uses RBCE as output data. The Random Forest model is built via Matlab software. In model parameter settings, the original sample data is split into 80 training set entries and 19 testing set entries. The decision tree count is set to 85, and the minimum leaf tree size to 3. After multiple trainings, a well fitted Random Forest model is obtained. The model with an *R*^2^ of 0.91754 is chosen as the final model due to its good fitting performance (Figure 7).

**Figure 8:**
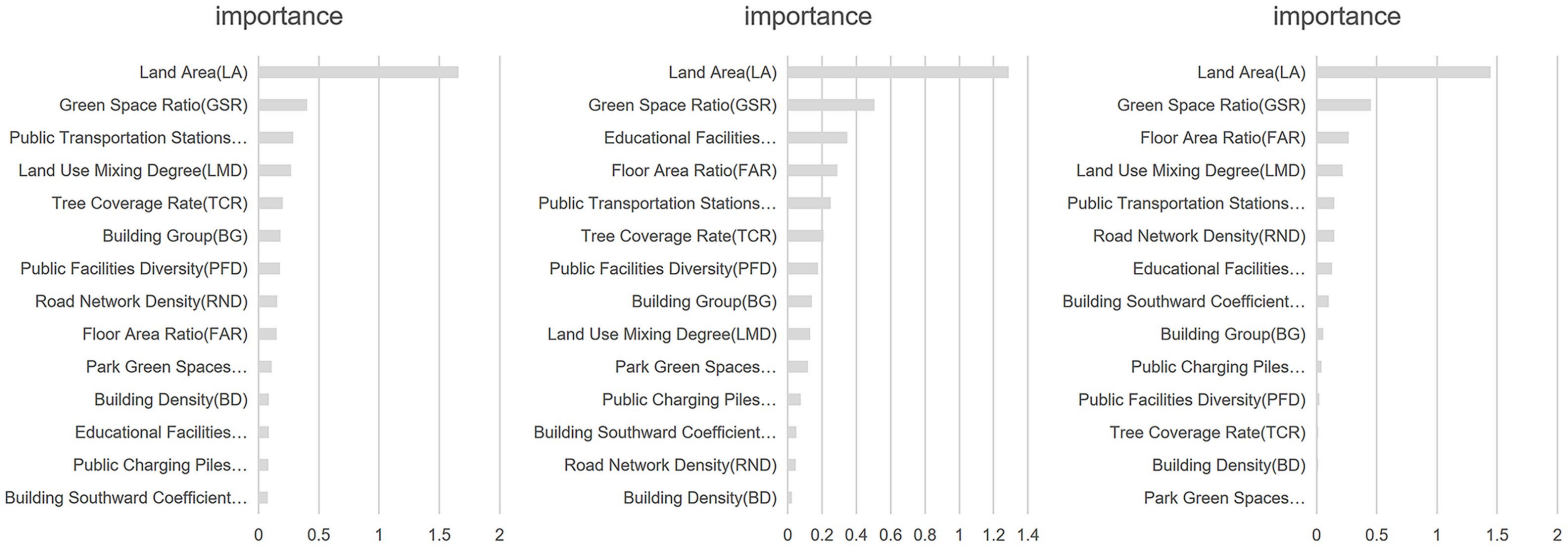
The importance of the impact of BEF on RBCE in 2021–2023.

Based on the constructed Random Forest model, the significance of the impact of 14 BEF on the RBCE in Hongqiao District, Tianjin, was determined, as shown in Figure 8. The fitting results indicate that these BEF can be divided into four groups based on their importance. The most critical factor is LA, which has the greatest impact on RBCE. Second are the LMD and GSR, with an importance of around 0.35, signifying a substantial influence. The importance of eight BEF, including BG, BSC, PFD, EFA, PCPA, RND, PTSD, and TCR, is basically distributed between 0.1 and 0.3. Their importance is relatively low, that is, the impact on the RBCE is also relatively low. Finally, there are three indicators: BD, FAR, and PGSA. Their importance is all below 0.1, and they have the least impact on the RBCE.

Overall, among the BEF in Hongqiao District, Tianjin, the factors at the LU level have the greatest impact on the RBCE, with a total importance of 1.9815. Secondly, at the EC level and the RT level, their total importance is 0.6607 and 0.5116 respectively, and they have a greater impact on the RBCE. The total importance at the FC level is 0.375. The impact on the RBCE is relatively small. Among the five levels, the overall importance of the ASL level is the lowest, and its impact on the RBCE in Hongqiao District, Tianjin is the least.

## 4 Discussion

### 4.1 Comparison with existing methods

#### 4.1.1 Comparison with existing carbon emissions measurement method

Based on the RBCE measurement method proposed in this study, the electricity consumption of RB is calculated through information such as user names, electricity usage locations, electricity consumption, and voltage in AMI data, and then the RBCE is calculated. Due to the accurate positioning, high timeliness and high coverage of AMI data, it can well make up for the shortcomings of traditional data collection that consume a lot of human and material resources, and overcome the problems of low data accuracy, poor timeliness, lack of representativeness and lack of authenticity in traditional methods (33). Compared with the traditional RBCE measurement methods, this study proposes a RBCE measurement method framework based on AMI data, which has the advantages of strong representativeness, accurate scale and wide scope. In 2024, the spatial allocation model was used to study urban construction land carbon emissions (34). Compared with the RBCE measurement method framework based on AMI data proposed in this paper, this study can achieve the accuracy of RBCE at the block level and greatly improve the timeliness, representativeness and realism. The remaining studies, based on the statistics of energy consumption and population in the statistical yearbook, were used to estimate the RBCE (35). Questionnaire survey data, and energy consumption simulation data all have the same problem (36). Therefore, compared with traditional carbon emission estimation methods, this method has the advantages of strong representativeness, accurate scale and wide range.

#### 4.1.2 Comparison with existing research

**Figure 9:**
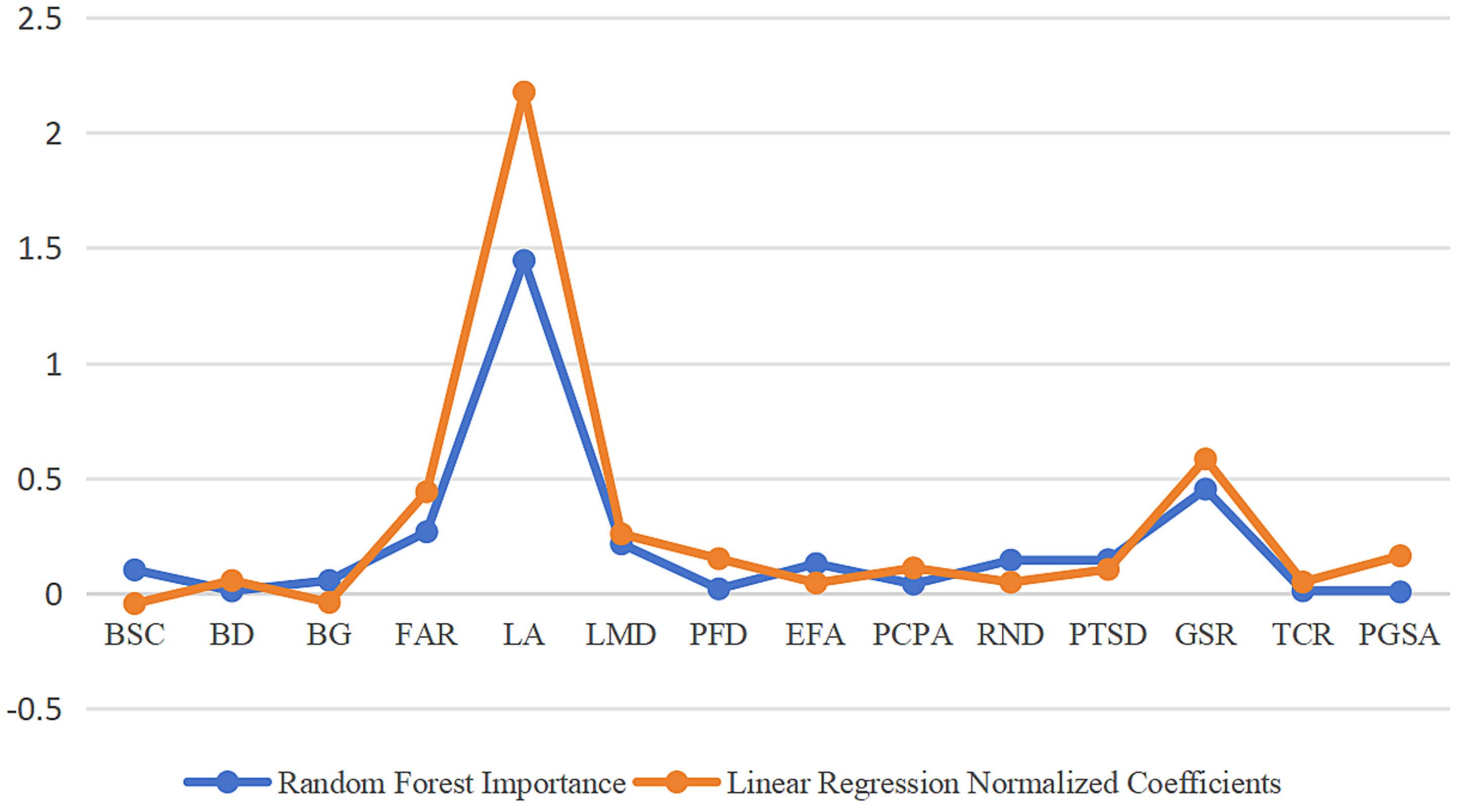
Comparison between Random Forest model and multiple regression standardized coefficient method.

For the ranking results of the BEF of the Random Forest analysis, the analysis results of this study were compared with the standardized coefficient method of multiple regression. The detailed differences between the two methods are shown in Figure 9. The comparison shows that the influence patterns of 14 BEF on RBCE present a certain degree of consistency. The key driving factors, such as LA, FAR, and LMD, rank among the top few in terms of their impact on RBCE in both methods, while PGSA and TCR rank among the bottom two in terms of their impact on RBCE, verifying the robustness of the core influence mechanism. However, Random Forests demonstrate more refined resolution in the identification of nonlinear relationships. For example, the importance ranking of the core variable GSR in the Random Forest (2nd place) is significantly higher than its position in the regression model (5th place), suggesting that this factor may have a threshold effect or interaction with other variables. Meanwhile, the analysis results in this study are more consistent with those in existing studies. However, the area density index RND showed low statistical significance in the regression (ranked 13th), but its Random Forest importance was relatively high (ranked 6th), reflecting that the linear model might have overestimated its independent contribution. Relevant studies have also confirmed this. This difference essentially stems from the Random Forest’s ability to capture complex interaction effects and asymmetric relationships, complementing the limitations of traditional regression in explaining high-dimensional nonlinear systems. The estimation results of this study are more accurate at the plaque level and can better support urban planning at medium and small scales.

### 4.2 Robustness assessment

Despite the limitations of the time scale, this study adopted the following methods to evaluate and enhance the robustness of the research results. Firstly, cross-year model consistency check. This study calculated the spatial distribution of RBCE and the regression coefficients of key driving factors for each year of 2021, 2022, and 2023, respectively. Analysis shows that despite annual fluctuations, core findings such as the spatial agglomeration characteristics of high-emission areas, the position of LA as the primary driving factor, the significant inhibitory effect of GSR, and the positive emission reduction value of LMD remain relatively consistent in the 3 years. In addition, the Moran’s I value of the spatial autocorrelation index is significant and stable, which is 0.275, respectively. 0.291 and 0.288. Secondly, verification of model stability. When constructing a high-precision Random Forest model, the study strictly adopted cross-validation, especially time series cross-validation or annual validation techniques, to evaluate the predictive performance and stability of the model. The model performs stably and has controllable errors on the validation set, with R^2^ all greater than 0.9, indicating that the relationships captured by the model are reliable during the observation period. Thirdly, the degree of conformity with existing theories and literature. Based on the comparative analysis in 4.1, it can be known that the driving factors discovered in this study are highly consistent with the theoretical basis of urban morphology, environmental science, and energy geography, as well as the conclusions of many related empirical studies. For example, based on the measured data of spatial morphological parameters of six local climate zones in Guangzhou, Liu et al. proposed a parametric optimization design technology for high-density residential communities based on the Dynamic Local Energy Balance (DLEB) model, and formed a perimetry oriented spatial optimization strategy for high-density residential communities. The suggested building density is 0.45–0.5, and the number of building floors is 10–20. The contribution rates of building floors and density to the comprehensive indicators are 48 and 43% respectively, thereby providing theoretical and technical guidance for the construction of dense residential areas (37). This consistency also indirectly supports the credibility of the findings of this study. Finally, data quality and granularity. The research utilized high-precision AMI data from State Grid (rather than estimated data) and conducted analyses on fine micro-scale RBs (99 blocks), which helped reduce data errors and the uncertainty brought about by scale effects, thereby providing more reliable basic information within the available data range.

### 4.3 Effects of BEF

#### 4.3.1 Effects of the overall block-scale

Based on the spatial distribution characteristics and the results of autocorrelation analysis, this study found that the high-emission areas show significant spatial agglomeration and infectivity, and are mainly distributed in the west-south direction of the study area. Based on the analysis of key driving factors, we believe that the formation of this spatial pattern is the result of the combined effect of BEF at the block scale. On one hand, the west-south area is home to mature RBs with relatively high FAR and LA, but low GSR and LMD. The BD and activity intensity in these areas may be higher, leading to concentrated energy demand and a lack of effective ecological buffers. At the same time, a single function may increase indirect emissions such as commuting. The peripheral low-carbon zones may benefit from relatively loose development densities, higher GSR, and possible mixed functions, or represent newer development projects that have adopted certain energy-saving standards. On other hand, the observed spatial accumulation of high-emission areas indicates the mutual influence among blocks, not only intensifying climate risks but also threatening residents’ health through an “exposure chain reaction,” triggering multiple health crises. This might stem from: (1) Similar development models and eras. Adjacent blocks may adopt similar planning standards and building codes, resulting in similar energy demand structures. (2) Infrastructure sharing and spillover effects. High-emission RBs may concentrate high-energy-consuming public facilities or commercial activities, and their impact may radiate to adjacent RBs. (3) Proximity of socio-economic characteristics. Residents’ income, consumption habits, commuting patterns, etc. may have spatial continuity, leading to similarities in energy usage patterns. (4) Local climate effects. Such as the urban heat island effect, may be more significant in high-density built-up areas and affect the surrounding areas. This contagiousness emphasizes that block planning cannot be carried out in isolation and needs to consider the impact of neighboring areas and regional collaborative emission reduction and health strategies.

#### 4.3.2 Effects of synergy of multiple factors

LA is the most significant positive driver of RBCE, while GSR is the most significant negative inhibitory factor. It is worth noting that the inhibitory effect of GSR exceeds the traditional high-intensity development indicators, highlighting the crucial role of ecological spaces in reducing emissions in micro-scale RBs. More importantly, there may be a strong antagonistic effect between LA and GSR. In larger RBs in LA, there is usually more building coverage and hardened surfaces, which weakens the space available for greening and thus limits the emission reduction benefits of GSR. Conversely, in RBs where LA is restricted, enhancing GSR through meticulous design, such as vertical greening and pocket parks, may become a key strategy to counteract the negative effects of high-density development, and may even generate a synergistic effect. For example, high GSR can not only squeeze carbon and release oxygen, but also alleviate the heat island effect through cooling, indirectly reducing the energy consumption for building cooling. In densely populated areas, this indirect emission reduction benefit may be magnified and can significantly promote the health of residents. For instance, it significantly reduces the risks of asthma, ischemic heart disease and stroke. The visibility and accessibility of this green space also significantly lower the levels of anxiety and depression, etc. Secondly, LMD also demonstrates value in reducing emissions. Its mechanism of action may lie in reducing residents’ reliance on motor vehicles, shortening commuting distances, and thereby lowering indirect emissions related to transportation. The role of LMD may complement or coordinate with that of LA and GSR. For example, in a neighborhood with a certain LA, a higher LMD can optimize the internal travel structure. A better GSR can enhance the livability of the mixed-function environment, attract residents to move more within the block, and further strengthen the emission reduction effect of LMD. Future research needs to quantify the interaction effects among multiple elements more precisely, for instance, by introducing interaction terms or more complex model structures. Based on the above analysis, it is helpful to propose more targeted and integrated planning strategies. When controlling the expansion of LA, it is necessary to enhance GSR in conjunction. In the planning of new districts, emphasis should be placed on the collaborative design of LMD and GSR. Formulate regional coordinated emission reduction plans for high-emission clusters, etc. It is suggested that future research should use models containing interaction terms, structural equation models or machine learning interpretability tools (such as SHAP value interaction analysis) to quantitatively reveal these complex combined effects.

#### 4.3.3 Effects of single-dimensional factors

**Figure 10:**
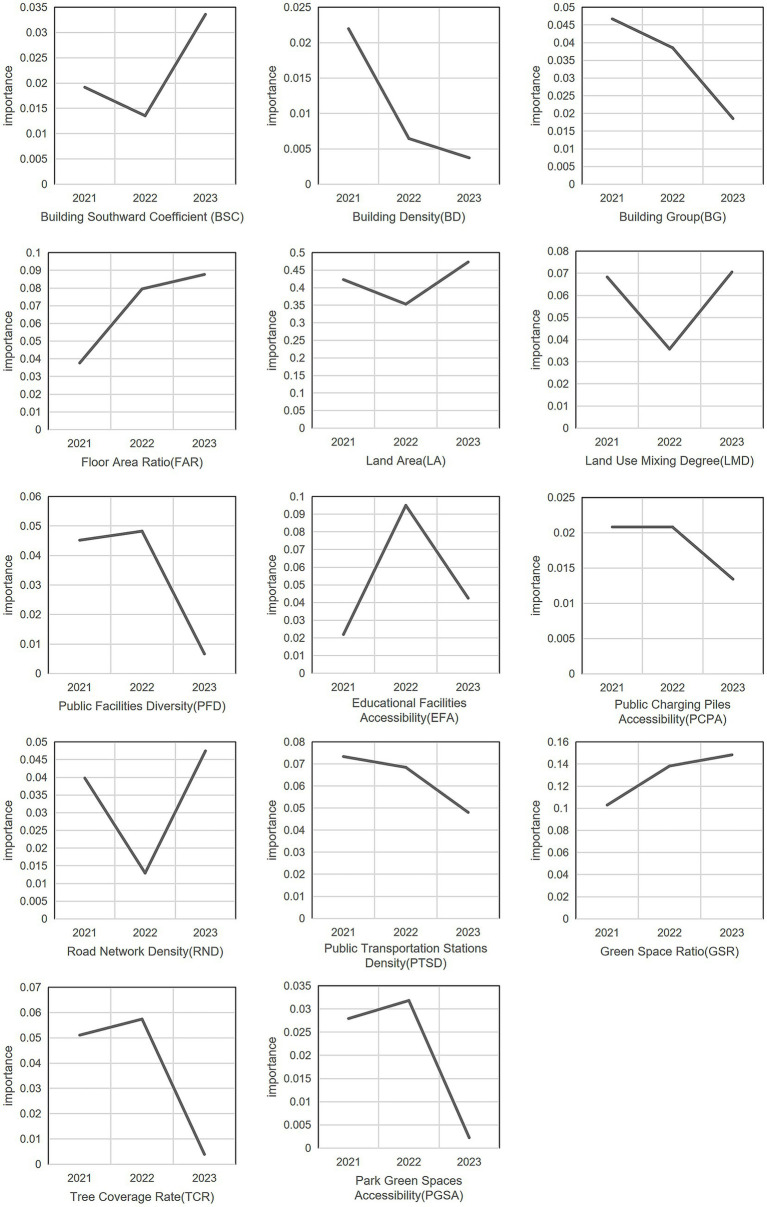
The impact of 14 BEF on RBCE from 2021 to 2023.

The above results indicate that different types of BEF lead to significant differences in RBCE. However, based on the quantitative results, the influence mechanism of BEF on RBCE still needs to be further explored to provide effective and scientific planning suggestions at the urban design level. The study conducted a separate analysis of the impact of 14 BEF on RBCE from 2021 to 2023 (Figure 10).

##### 4.3.3.1 Architectural spatial layout

The BSC, BD, and BG are the three main factors describing the ASL. Among them, the significance of the impact of BG on the RBCE is 0.18, which is higher than that of BD and the BSC. Different forms of BG not only have a direct impact on the energy consumption of buildings within the region, but also affect the energy consumption behavior of individual buildings within the region through changes in the external physical environment of the buildings, thereby achieving the influence on the energy consumption of buildings within the region. These intermediary channels mainly include the influence of urban spatial forms on the urban heat island effect, urban wind environment, and building lighting. Although limited by data, the study recognizes that the heterogeneity of mixed building types is crucial. High-rise buildings usually have lower per capita energy consumption for heating and cooling, but they may have higher energy consumption in public areas (38). The per capita energy consumption for heating and cooling in low-rise buildings is usually higher, but the public energy consumption is lower. In mixed RBs, the proportion and spatial layout of different types of buildings can affect the overall energy demand structure of the block, microclimate, and residents’ behaviors. Future studies need to integrate detailed data on building types and spatial distribution to more precisely quantify the impact of this heterogeneity on carbon emissions (39). Similarly, there exists the same logical correspondence between the BSC, BD and RBCE. Some studies have also proved this point. In this study, BD was significantly negatively correlated with RBCE. On one hand, BD specifically reflects the spatial distribution characteristics of building entities on the unit residential block, as it affects the formation of the wind, heat and light environment on the residential block and further has an important impact on carbon emissions (40). On other hand, high BD means an increase in building coverage, which will increase the mutual occlusion between buildings to a certain extent, thereby affecting the utilization of solar energy in winter and increasing the energy consumption for building heating (41).

##### 4.3.3.2 Land use

The FAR, LA, and LMD are the three main factors describing LU. Among them, the significance of LA on the RBCE is 1.66, which is higher than that of FAR and LMD. This is because as LA increases, the building area of RBs also increases accordingly. Building area, as a symbol of urban spatial intensity, is usually proportional to it (42). Liu et al. conducted an empirical study on the relationship between RBCE and LA in Changxing, China, using the simulation data of ENVI-met. The results show that in the larger suburbs of LA, the RBCE is higher, while in the smaller central urban area of LA, the RBCE is lower (43). The LMD mainly refers to the proportion of mixed building areas of other functions on a single type of construction land. The influence of LMD on RBCE is mainly reflected in the energy consumption usage of different types of buildings such as lighting, heating, and cooling in residential buildings, as well as the energy consumption usage of lighting, heating, cooling, and office equipment in public buildings. Due to the significant differences in the intensity and density of internal personnel activities among buildings with different functions, there are considerable variations in building energy consumption (44). FAR is an important BEF that affects RBCE. Although it was not directly included as a three-dimensional morphological indicator in this study, many literatures have confirmed its key role. For example, the FAR directly affects the volume ratio of building surface area, and thereby influences the heat exchange efficiency. High-intensity development is usually associated with a lower sky view factor (SVF), which limits the sky view, affects long-wave radiative cooling and natural lighting, and may increase the demand for artificial lighting and air conditioning. Additionally, the intensity of building development directly affects the wind environment and shading, which in turn influences the overall energy consumption performance of the building complex. Based on these theoretical mechanisms, we can speculate that a larger FAR may be associated with higher development intensity and potentially higher building volume, and its underlying physical mechanism is likely to be achieved through changes in three-dimensional form and microclimate. For example, the promoting effect of FAR may partly stem from its ability to allow for larger building volumes and more compact layouts (45). Future research must prioritize the integration of precise three-dimensional morphological data to more comprehensively reveal the multi-dimensional path by which BEF affects RBCE.

##### 4.3.3.3 Facility configuration

The PFD and EFA are two main factors for describing FC. Among them, the importance of the EFA on the RBCE is 0.09, which is higher than that of the PFD. This is because the distribution of EFA is an important factor in urban planning. Both the layout of urban residential block and the planning of road systems will be influenced by the layout of educational facilities. For example, the suburbanization development model of cities in the United States has led to the disorderly spread of cities along highways, mainly manifested in aspects such as excessively low urban construction density, scattered population residence, inconvenient life, residents’ excessive reliance on private cars for travel leading to the deterioration of environmental problems, the lack of community culture, and the waste of land resources. This suburban sprawling development pattern has had a negative impact on students’ school enrollment, resulting in an increase in the service radius of primary schools. More and more parents use private cars to pick up and drop off their children at school, which increases the carbon emissions of cars during the process of picking up and dropping off students. Some studies have also proved this point. Furthermore, as an important BEF, the PFD is also closely related to the RBCE. Apart from education, the PFD covers multiple aspects such as transportation, energy supply, medical care, and leisure and entertainment. The richness and layout rationality of these facilities have a profound impact on residents’ daily life behavior patterns and carbon emission levels. If the layout of public service facilities is unreasonable, overly concentrated or scattered, it may also lead to residents having to walk a longer distance when using these facilities, thereby increasing carbon emissions.

##### 4.3.3.4 Road traffic

The PCPA, RND, and PTSD are the three main factors describing RT. Among them, the importance of the impact of PTSD on the RBCE is 0.29, which is higher than that of PCPA and RND. On one hand, the higher the density of PTSD is, the shorter the walking distance for residents to reach the stops will be, and the convenience of travel will be significantly improved. When the PTSD is high enough, the possibility for residents to choose public transportation in their daily trips will increase significantly. For instance, in areas with a high density of bus stops, residents can walk to the nearest bus stop or subway station in a short time without relying on high carbon emissions vehicles such as private cars or motorcycles. This shift in travel mode directly reduces carbon emissions caused by individual driving. Studies show that for every increase in the density of stations by a certain proportion, the proportion of residents choosing public transportation for travel will also increase accordingly, thereby effectively reducing the per capita carbon emission level. On other hand, the increase in the PTSD also has a positive impact on the urban spatial structure, and thereby indirectly affect carbon emissions. A public transportation network with dense stations can guide the rational layout of urban space and promote the compact development of the city. Additionally, the indicators for measuring road traffic also include PCPA and RND. In a certain area, the higher the RND and the smaller the block size, the smaller the LA. Small scale land can offer a variety of travel options, increase pedestrian accessibility, and reduce carbon emissions from transportation energy consumption. The increase in the number of PCPA can encourage residents to use more new energy vehicles for travel, thereby reducing carbon emissions.

##### 4.3.3.5 Ecological construction

The GSR, TCR, and PGSA are the three main factors describing EC. Among them, the significance of the GSR on the RBCE is 0.403, which is higher than that of the TCR and the PGSA. The GSR refers to the proportion of the area of various types of green spaces (including parks, squares, street green Spaces, protective green Spaces, affiliated green Spaces, etc.) to the total area of a certain region. The level of GSR has a significant impact on RBCE. On one hand, green spaces are important carbon sink resources in urban ecosystems. Plants absorb CO_2_ and release O_2_ through photosynthesis. This process can effectively reduce the concentration of CO_2_ in the atmosphere, thereby lowering carbon emissions. Trees, grasslands, and other vegetation in green spaces absorb a large amount of CO_2_ during their growth process and fix it in their bodies, forming carbon sinks. For example, the research conducted by Vaccari in Italy on the city of Florence shows that within the 102.3 km^2^ of the city, 29.1 km^2^ of green space offset 6.2% of the direct carbon emissions, and proposes a low carbon model for spatial organization based on suburban green rings (46). On other hand, in terms of TCR and PGSA, the carbon sink capacity of green spaces not only depends on their area but is also closely related to the type and quality of vegetation. For example, the carbon sink capacity of forest green spaces is usually higher than that of grasslands, and the carbon absorption capacity of trees is higher than that of shrubs and herbaceous plants. Therefore, while increasing the GSR, optimizing the vegetation structure and improving the quality of vegetation can further enhance the carbon sink function of green spaces and effectively reduce carbon emissions.

### 4.4 Limitations and further improvements

In recent years, how accurately analyzing the effects of BEF on RBCE has been a great concern. The results obtained in this study can be used to supplement and improve the control index system of the existing urban planning at the medium and micro scales. This is of great significance for guiding policymakers to formulate targeted emission reduction policies or helping urban planners to formulate low-carbon urban planning schemes that are more targeted to control RBCE.

There are also some uncertainties in this study. Firstly, although the research methods we proposed provide a new possibility to explore the effects of BEF on RBCE under the condition of limited statistical samples, there are only 99 residential blocks samples, which will affect the accuracy of the results. Secondly, compared with other studies, the RBCE is represented by electricity consumption carbon emissions in this study, without considering the gas, transportation, and waste transfer. Although it is feasible, it will also have errors. Thirdly, the time series of building energy consumption data needs to be expanded. The robustness of the results of this study can be enhanced by analyzing the changes in panel data over the years. As a long-term study, with the continuous update of subsequent data, observations from 2024 and beyond will be included to further verify the long-term resilience of BEF on RBCE. However, the driving mechanisms and spatial differentiation patterns discovered in this study provide immediate evidence for the low-carbon renewal of residential blocks. Finally, due to the limitations of the current studies data foundation, the BEF system adopted in this study mainly focuses on the two-dimensional land use and planar morphological characteristics at the block scale, and fails to deeply depict the spatial distribution heterogeneity of building types within the block (for example, the mixed proportion and spatial combination pattern of low-rise villas and high-rise apartments) and the quantitative characterization of three-dimensional morphological characteristics. It is only to conduct in-depth explanations from the perspectives of theoretical mechanisms and existing literature evidence during the discussion. This heterogeneity and three-dimensional elements also affect to a certain extent the solar radiation acquisition, natural ventilation potential and thermal environment comfort within the block, resulting in significant differences in energy consumption intensity and carbon emission patterns among different building units within the same block, thereby influencing the accuracy of the overall emission assessment of the block and the depth of mechanism explanation.

Future studies on low carbon RBs can become more detailed, refined and goal-oriented, and clearly incorporate health risk mitigation targets. Conduct targeted research and assessment for each RB type or individual area. Based on the influencing factors of carbon emissions related to residential electricity usage and the local BEF situation, provide targeted strategic suggestions. Firstly, low carbon RB planning research is adopting more methods, which demand more of research data. Internet of Things and big data technologies can fill this gap. In future research, more accurate and timely data will be obtained from multiple sources, and artificial intelligence will be utilized to process and analyze the data. If higher-resolution data (such as building census data, street view image recognition, LiDAR point cloud classification) are integrated, and the heterogeneity of building types and more three-dimensional morphological indicators (such as building height, volume, SVF, H/W and other key three-dimensional parameters) are incorporated into the BEF indicator system, Combined with more refined coupling models (such as combining microclimate simulation ENVI-met and energy consumption simulation), quantitatively analyze its influence path. Clarify the complete impact pathways of BEF on the microenvironment, carbon emissions, and residents’ exposure to air pollutants and health risks (such as cardiopulmonary diseases and neurological impacts). Secondly, factors in low carbon RB studies should extend beyond material and spatial BEF. Explore and analyze the living habits and concepts of people with different regional and cultural characteristics, as well as their impact on carbon emissions and indoor and outdoor environmental health risks (such as increased emissions and heat-related disease risks due to excessive reliance on air conditioning). Thirdly, Strengthen the promotion of low-carbon concepts, enhance residents’ awareness and participation, and guide the formation of daily behavioral patterns that not only reduce carbon footprints but also improve micro-environmental health (such as encouraging natural ventilation and reducing the use of highly polluting fuels). Additionally, the government should establish and improve technical standards, policies and regulations for low-carbon residential areas, and clearly incorporate the health synergy benefits such as reducing local pollution exposure and alleviating the heat island effect as core goals into the norms and assessment system. Standardize its construction and optimization to provide strong technical and policy support for the low-carbon development of residential buildings. Ensure the technical feasibility of the policy and maximize the health benefits.

## 5 Conclusion

This study took 99 RBs in Hongqiao District, Tianjin as the research objects, systematically analyzed the temporal evolution and spatial distribution characteristics of RBCE during the period from 2021 to 2023, and constructed an index evaluation system. The evaluation indicators were fitted through the Random Forest model, revealing the impact of the BEF on the RBCE. The main conclusions are as follows:

(1) The study proposed a fine analytical framework for the microscale of RBCE. This study focuses on the micro-spatial unit of RBs, systematically revealing the uniqueness of Hongqiao District, Tianjin in terms of temporal dynamics (relatively stable but fluctuating from 2021 to 2023) and spatial patterns. Not only were the high-value and low-value areas of total RBCE and RBCEI identified, but more importantly, through spatial autocorrelation analysis, it was confirmed at the block scale that RBCE has a significant global spatial positive correlation agglomeration pattern (agglomeration on the west and south sides).
(2) Accurately identify and quantify the key driving factors of the BEF and their hierarchical relationships. Using the high-precision Random Forest model (with a goodness of fit R^2^ as high as 0.91754), the impact intensity of up to 14 BEF on RBCE was systematically quantified and evaluated, and their importance levels were clearly ranked. The LA has been identified as the absolute primary driver influencing RBCE, and its significance far exceeds that of all other factors. This indicates that merely focusing on the architectural form or density might be biased. Controlling the overall land expansion of the block is the most crucial entry point for emission reduction. Most importantly, the GSR, as a core indicator characterizing the ecological attributes of a block, ranks second in importance, significantly higher than traditional high-intensity development indicators, as well as BG and RND. This strongly confirms that EC elements have a prominent and independent negative impact on reducing RBCE at the micro scale, providing direct empirical support for the application of the “ecological carbon sequestration” concept in block planning. Furthermore, LMD has also been identified as a key factor with significant potential for emissions reduction.

This study not only provides a more powerful methodological toolkit for understanding the complex interactions between humans and the land system, but also offers evidence-based precise intervention strategies for low-carbon and public health-oriented residential block planning, and provides clear, actionable and prioritized practical guidelines for low-carbon planning and renovation at the RBs level, considering mental health, respiratory and cardiovascular protection.

## Data Availability

The raw data supporting the conclusions of this article will be made available by the authors, without undue reservation.
